# Supplementary material for: A non-B DNA binding peptidomimetic channel alters cellular functions
Source: Nat Commun. 2024 Jun 20;15:5275. doi: 10.1038/s41467-024-49534-0 (PMC11190219; doi:10.1038/s41467-024-49534-0)
Supplement: Supplementary file 1 — Supplementary Information [file 41467_2024_49534_MOESM1_ESM.pdf]

Supplementary Information

Expanding the landscape of ion transporters: A non-B DNA binding peptidomimetic channel alters cellular functions

Raj Paul, Debasish Dutta, Titas Kumar Mukhopadhyay, Diana Müller, Binayak Lala, Ayan Datta, Harald Schwalbe, Jyotirmayee Dash*

1.0 General information S2
2.0 Synthesis of thiazole based peptidomimetics S3
3.0 NMR spectra of compounds S9
4.0 HPLC analysis S18
5.0 Lipids, oligonucleotides and chemicals S18
6.0 TEM imaging S18
7.0 Ion transport study by fluorescence spectroscopy S19
7.1 Preparation of large unilamellar vesicles (LUVs) S19
7.2 Monitoring ion transport by HPTS assay S19
7.3 Cl^- transport by lucigenin assay S22
7.4 Preparation of carboxyfluorescein (CF) encapsulated vesicles S22
7.5 Carboxyfluorescein release experiments S22
8.0 Formation of giant unilamellar vesicles (GUVs) S23
9.0 Confocal imaging in GUVs and cells S23
10.0 Safranin O assay S24
11.0 Conductance measurements by patch-clamp technique S25
12.0 Dynamic light scattering S25
13.0 Computational details and molecular dynamics simulation S26
14.0 Cell culture S31
15.0 Intracellular Na^+ or K^+ concentration measurement S31
16.0 Cell growth inhibition assay S31
17.0 Immunofluorescence via confocal microscopy S32
18.0 Quantitative real-time PCR S33
19.0 Dual luciferase reporter assay S36
20.0 UV absorption spectroscopy S37
21.0 Melting experiments by FRET S38
22.0 Fluorescence spectroscopy S39
23.0 NMR titration S42

1.0 General information

All experiments were carried out under an inert atmosphere of argon in flame-dried flasks. Solvents were dried using standard procedures. All starting materials were obtained from commercial suppliers and used as received. Products were purified by flash chromatography on silica gel (100-200 mesh, Merck). Unless otherwise stated, yields refer to analytical pure samples. Melting points were measured with BÜCHI Melting point B-545 and are uncorrected. NMR spectra were recorded in $CDCl_3$ unless otherwise stated. **^{1}H NMR** spectra were recorded at 500 MHz using Brüker AVANCE 500 MHz and JEOL 400 MHz instruments at 298 K. Signals are quoted as δ values in ppm using residual protonated solvent signals as internal standard ($CDCl_3$: δ 7.26 ppm). Data is reported as follows: chemical shift, integration, multiplicity (s = singlet, d = doublet, t = triplet, q = quartet, p = pentet, br = broad, m = multiplet), and coupling constants (Hz). **^{13}C NMR** spectra were recorded on either a JEOL-400 (100 MHz), or a Brüker AVANCE 500 MHz (125 MHz) with complete proton decoupling. Chemical shifts (δ) are reported in ppm downfield from tetramethylsilane with the solvent as the internal reference ($CDCl_3$: δ 77.16 ppm). Infrared (FTIR) spectra (ν_{max}) are recorded on a Perkin Elmer spectrophotometer Spectrum RX1 using KBr disk techniques for solid compounds and as a thin film (neat) for liquid samples and are reported in cm^{-1}. **HRMS** analyses were performed with Q-TOF YA263 high resolution (Water Corporation) instruments by +ve mode electrospray ionization.

2.0 Synthesis of thiazole based peptidomimetics

Dimeric thiazole peptidomimetic was synthesized using Cu(I) catalyzed azide–alkyne cycloaddition as depicted in Scheme S1. Bis-thiazole acid **S6** was prepared in four steps from ethyl 2-aminothiazole-4-carboxylate **S1** and 5-hexynoic acid **S2** by stepwise amide coupling using HBTU (hexafluorophosphate benzotriazole tetramethyl uronium) as a coupling reagent and followed by ester hydrolysis using LiOH. The amide coupling between ethyl 2-aminothiazole-4-carboxylate **S1** and 5-hexynoic acid **S2** was carried out using HBTU in presence of DIPEA (di-isopropyl-ethyl amine) as a base to afford thiazole derivative **S3** which upon ester hydrolysis using LiOH provided the corresponding acid **S4**. Thiazole acid **S4** was next coupled with **S1** using HBTU in the presence of DIPEA and a catalytic amount of DMAP (4-(dimethylamino)pyridine) to provide thiazole peptide **S5** which underwent ester hydrolysis to provide the acid **S6**. The amine side chain was incorporated by amide coupling of **S6** with 3-(dimethylamino)-1-propylamine **S7** resulting in bis-thiazole alkyne **2**.

The thiazole azide **1** was synthesized by the coupling between Boc protected thiazole acid S**8** and 2-azidoethylamine **S9**. The cycloaddition reaction between alkyne functionalized bis-thiazole peptide **2** and azido functionalized bis-thiazole peptide **1** was carried out in the presence of copper sulphate and sodium ascorbate to provide the desired thiazole peptide **TBP1** and subsequent Boc-deprotection provided the dimeric thiazole dipeptide **TBP2** in high overall yield.

i) Synthesis of thiazole alkyne:

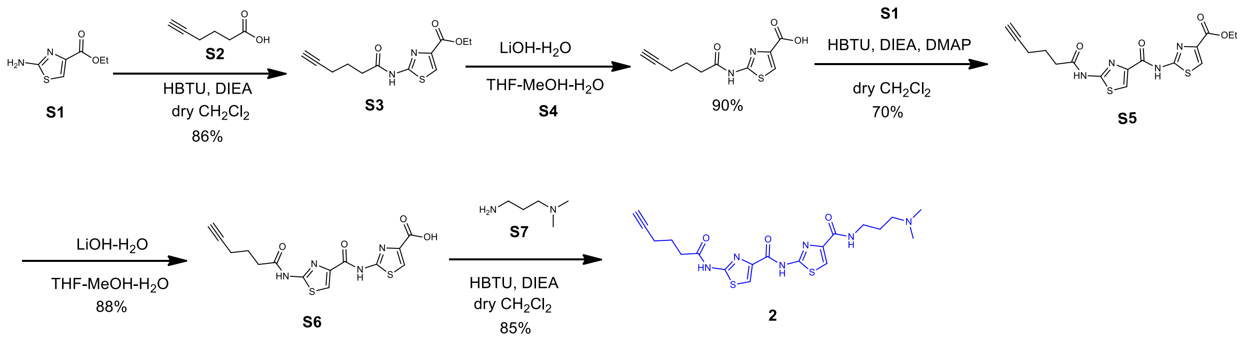

ii) Synthesis of thiazole azide:

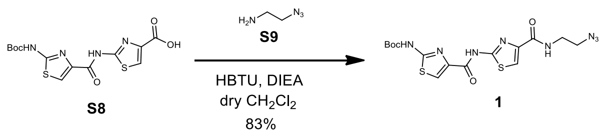

iii) Synthesis of dimeric thiazole peptide TBP2:

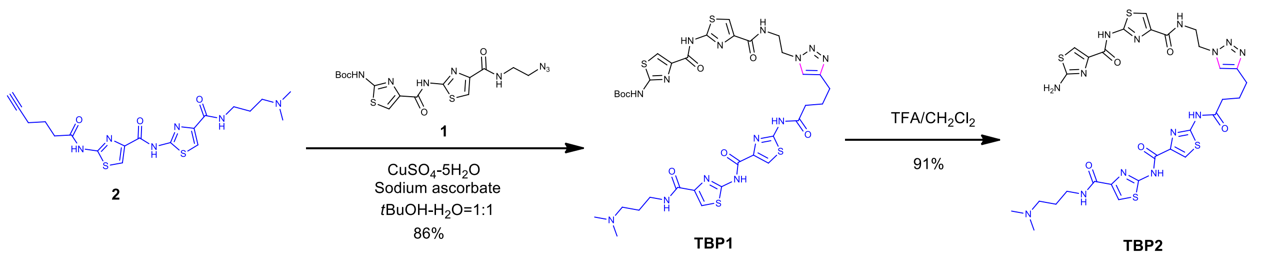

Fig. S1: Synthesis of dimeric thiazole compounds.

Synthesis of thiazole peptide S3: To a stirred solution of thiazole amine **S1** (2.0 gm, 11.61 mmol) in dry CH_2Cl_2, DIEA (6.07 mL, 34.81 mmol) was added to it followed by the addition of 5-hexynoic acid **S2** (1.28 mL, 11.61 mmol). After stirring for 15 minutes at 0 °C, HBTU (6.6 gm, 17.41 mmol) was added. The reaction was allowed to stir for 24 hours at room temperature. Subsequently, the reaction mixture was concentrated and the residue was dissolved in ethylacetate (3×10 mL). The ethylacetate layer was successively washed with 1(N) HCl solution (3×10 mL), saturated $NaHCO_3$ solution (3×10 mL) and brine. After drying with Na_2SO_4 and filtration, the solvents were removed under vacuum. The residue was purified by column chromatography to furnish the desired compound **S3** (2.66 gm, 86 %) as a white solid; ^1H NMR (500 MHz, $CDCl_3$): 10.87 (s, 1H), 7.82 (s, 1H), 4.36 (q, J = 7.0 Hz, 2H), 2.63 (t, J = 7.4 Hz, 2H), 2.28 (td, J = 6.8, 2.5 Hz, 2H), 1.94 – 1.90 (m, 3H, merged with CH_2 peak), 1.37 (t, J = 7.0 Hz, 3H); ^{13}C NMR (100 MHz, $CDCl_3$): 171.4, 161.3, 159.1, 141.1, 122.2, 83.0, 69.6, 61.6, 34.7, 23.5, 17.9, 14.4; HRMS (ESI) calcd for $C_{12}H_{14}N_2O_3SNa$ $[M+Na]^+$: 289.0623; Found: 289.0626.

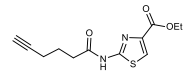

Synthesis of thiazole acid S4: Using general procedure **GP-1**, LiOH-H_2O (1.18 g, 28.16 mmol) and thiazole peptide **S3** (2.5 g, 9.39 mmol) in THF/MeOH/H_2O (20 mL) were stirred for 4 h to provide the corresponding thiazole acid **S4** (2.01 g, 90%) as a white solid; ^1H NMR (400 MHz, DMSO-d_6): 12.80 (s, 1H), 12.43 (s, 1H), 7.94 (s, 1H), 2.81 (t, J = 2.8 Hz, 1H), 2.53 (t, J = 7.6 Hz, 2H), 2.21 (td, J = 7.2, 2.8 Hz, 2H), 1.81 – 1.73 (m, 2H); 13C NMR (100 MHz, DMSO-d_6): 171.2, 162.3, 157.7, 141.9, 122.0, 83.6, 71.7, 33.6, 23.3, 17.2; HRMS (ESI) calcd for $C_{10}H_{10}N_2O_3SNa$ $[M+Na]^+$: 261.0310; Found: 261.0311.

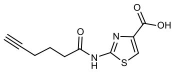

Synthesis of thiazole peptide S5: To a stirred solution of thiazole amine **S1** (1.08 gm, 6.29

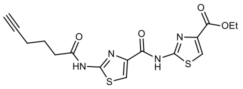

mmol) in dry CH_2Cl_2, DIEA (3.29 mL, 18.87 mmol) and DMAP (154 mg, 1.26 mmol) were added followed by the addition of thiazole acid **S4** (1.5 gm, 6.29 mmol). After stirring for 15 minutes at 0 °C, HBTU (3.75 gm, 9.435 mmol) was added. The reaction was allowed to stir for 24 hours at room temperature. The reaction mixture was then concentrated and the residue was dissolved in ethylacetate. The ethylacetate layer was successively washed with 1(N) HCl solution (3×10 mL), saturated $NaHCO_3$ solution (3×10 mL) and brine. After drying with Na_2SO_4 and filtration, the solvents were removed under vacuum and purified by column chromatography to provide the desired compound **S5** (1.72 gm, 70 %) as a white solid; ^{1}H NMR (500 MHz, DMSO-d_6): 12.5 brs, 2H), 8.31 (s, 1H), 8.10 (s, 1H), 4.29 (q, *J* = 6.8 Hz, 2H), 2.81 (t, *J* = 2.7 Hz, 1H), 2.57 (t, *J* = 7.4 Hz, 2H), 2.24-2.21 (m, 2H), 1.82 – 1.76 (m, 2H), 1.30 (t, *J* = 7.1 Hz, 3H); ^{13}C NMR (125 MHz, DMSO-d_6): 171.4, 160.9, 159.4, 158.0, 141.9, 141.2, 123.2, 120.6, 83.7, 71.7, 60.6, 33.7, 23.3, 17.2, 14.2; HRMS (ESI) calcd for $C_{16}H_{17}N_4O_4S_2$ $[M+H]^+$: 393.0686; Found: 393.0693.

Synthesis of thiazole acid S6: Using general procedure **GP-1**, thiazole peptide **S5** (1.5 g, 3.822

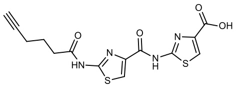

mmol) was stirred for 4 h in THF/MeOH/H_2O (20 mL) with LiOH-H_2O (482 mg, 11.47 mmol), , providing the corresponding thiazole acid **S6** (1.22 g, 88%) as a white solid; ^{1}H NMR (500 MHz, DMSO-d_6): 13.86 (s, 1H), 13.00 (s, 1H), 8.35 (s, 1H), 7.52 (s, 1H), 2.83 (t, *J* = 2.7 Hz, 1H), 2.65 (t, *J* = 7.4 Hz, 2H), 2.29 (td, *J* = 7.2, 2.7 Hz, 2H), 1.91 – 1.85 (m, 2H); ^{13}C NMR (125 MHz, DMSO-d_6): 171.2, 165.1, 158.8, 157.3, 156.9, 150.3, 141.6, 119.1, 114.7, 83.7, 71.5, 33.8, 23.4, 17.3; HRMS (ESI) calcd for $C_{14}H_{13}N_4O_4S_2$ $[M+H]^+$: 365.0373; Found: 365.0373.

Synthesis of thiazole peptide 2: To a stirred solution of thiazole acid **S6** (1.0 gm, 2.744 mmol)

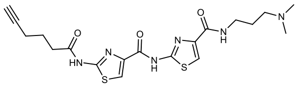

in dry CH_2Cl_2, DIEA (1.43 mL, 8.23 mmol) was added to it followed by the addition of amine **S7** (0.41 mL, 3.29 mmol). After stirring for 15 minutes at 0 °C, HBTU (1.56 gm, 4.12 mmol) was added. The reaction was stirred overnight at room temperature. After completion of the reaction, the reaction mixture was diluted with dichloromethane and the dichloromethane layer was successively washed with saturated $NaHCO_3$ solution (3×10 mL) and brine. After drying with Na_2SO_4 and filtration, the solvents were removed under vacuum and purified by column chromatography, provided the

desired compound **2** (1.05 gm, 85 %) as an off-white solid; ^{1}H NMR (400 MHz, $CDCl_3$): 8.47 (t, *J* = 6.0 Hz, 1H), 7.82 (s, 1H), 7.78 (s, 1H), 3.58 (q, *J* = 6.4 Hz, 2H), 2.84 (t, *J* = 7.2 Hz, 2H), 2.41-2.37 (m, 2H), 2.32 (t, *J* = 6.8 Hz, 2H), 2.07-2.01 (m, 9H, merged with one CH_2 and two CH_3 peaks), 1.81-1.74 (m, 2H); ^{13}C NMR (100 MHz, $CDCl_3$): 171.7, 164.1, 159.0, 158.7, 158.6, 145.2, 141.6, 121.1, 118.4, 83.4, 69.5, 56.8, 45.3, 38.0, 34.7, 27.1, 23.8, 18.1; HRMS (ESI) calcd for $C_{19}H_{25}N_6O_3S_2$ $[M+Na]^+$: 449.1424; Found: 449.1425.

Synthesis of thiazole peptide 1: To a stirred solution of thiazole acid **S8** (500 mg, 1.35 mmol)

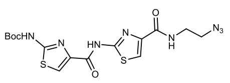

in dry CH_2Cl_2, DIEA (0.7 mL, 4.05 mmol) was added to it followed by the addition of azido amine **S9** (581 mg, 6.75 mmol). After stirring for 15 minutes at 0 °C, HBTU (767.5 mg, 2.025 mmol) was added. The reaction was stirred overnight at room temperature. The reaction mixture was subsequently diluted with dichloromethane. The dichloromethane layer was successively washed with saturated $NaHCO_3$ solution (3×10 mL) and brine. After drying with Na_2SO_4 and filtration, the solvents were removed under vacuum and purified by column chromatography to obtain the desired compound **1** (509 mg, 86 %) as a white solid; ^{1}H NMR (500 MHz, $CDCl_3$): 10.25 (s, 1H), 9.38 (s_{br}, 1H), 7.95 (s, 1H), 7.85 (s, 1H), 7.82 (s, 1H), 3.68 (q, *J* = 5.5 Hz, 2H), 3.57 (t, *J* = 5.5 Hz, 2H), 1.58 (s, 9H); ^{13}C NMR (100 MHz, $CDCl_3$): 162.5, 160.3, 158.6, 157.4, 152.6, 144.3, 142.1, 120.4, 118.9, 83.5, 51.1, 38.8, 28.3.

Synthesis of triazole containing thiazole peptide TBP1: Alkyne **2** (100 mg, 0.223 mmol) was dissolved in a 1:1 mixture of *t*BuOH/H_2O (3 mL). Copper(II) sulphate pentahydrate (5.6 mg,

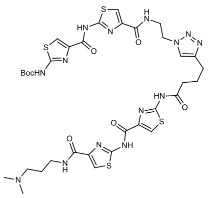

0.0223 mmol) and sodium ascorbate (8.5 mg, 0.0446 mmol) were added and the solution was stirred for 10 min. The azide **1** (117 mg, 0.2675 mmol) was added and the mixture was then allowed to stir for overnight. After completion of the reaction, the reaction mixture was concentrated. The crude product was purified by column chromatography to provide the desired peptide **TBP1** (170 mg, 86%) as an off-white solid; ^{1}H NMR (400 MHz, DMSO-d_6): 8.27 (t, *J* = 6.0 Hz, 1H), 8.21 (s, 1H), 8.16 (s, 1H), 8.09 (t, *J* = 6.0 Hz, 1H), 7.89 (s, 1H), 7.84 (s, 1H), 7.81 (s, 1H), 5.75 (s, CH_2Cl_2), 4.51 (t, *J* = 6.1 Hz, 2H), 3.72 (q, *J* = 6.0 Hz, 2H), 3.29 (q, *J* = 6.8 Hz, 2H), 2.67 (t, *J*= 7.56 Hz, 2H), 2.52 (t, *J* = 7.2 Hz, 2H, merged), 2.31 (t, *J* = 7.2 Hz, 2H), 2.18 (s, 6H), 1.97 – 1.89 (m, 2H), 1.69-1.62 (m, 2H), 1.50 (s, 9H); ^{13}C NMR (100 MHz, DMSO-d_6): 171.8, 160.9, 160.6, 160.2, 159.3, 159.2, 158.1, 157.6, 157.5, 153.0, 146.0, 144.9, 144.3, 142.4, 142.1, 122.3, 120.4, 120.3, 118.2, 117.5,

81.8, 56.7, 54.8 (CH_2Cl_2 peak), 48.6, 44.9, 37.1, 34.2, 31.1, 27.8, 26.9, 24.4, 24.3; HRMS (ESI) calcd for $C_{34}H_{43}N_{14}O_7S_4$ $[M+H]^+$: 887.2316; Found: 887.2 .

Synthesis of thiazole peptide TBP2: Boc protected thiazole peptide **TBP1** (100 mg, 0.113 mmol) was dissolved in CH_2Cl_2 (1 mL) and cooled to 0 °C. 1 mL Trifluoroacetic acid (equal amount as the solvent) was added and the solution was warmed to room temperature. The reaction mixture was stirred for about 3-4 hours at room temperature until starting material was fully consumed. The reaction was monitored by TLC. After completion of the reaction, the solvent was removed in vacuo and the residue was washed with ether. The solid residue was dried under vacuum to provide the corresponding amine free triazole containing thiazole peptide **TBP2** as a TFA salt (92.4 mg, 91%, white solid); ^{1}H NMR (400 MHz, DMSO-d_6): 12.37 (s, 1H), 11.65 (s, 1H), 11.15 (s, 1H), 9.58 (s, 1H), 8.34 (t, J = 6.0 Hz, 1H), 8.24 (s, 1H), 7.89 (s, 1H), 7.87 (s, 1H), 7.84 (s, 1H), 7.68 (s, 1H), 4.50 (t, J = 6.4 Hz, 2H), 3.71 (q, J = 6.0 Hz, 2H), 3.34 (q, J = 6.4 Hz, 2H), 3.11 – 3.05 (m, 2H), 2.78 (d, J = 4.4 Hz, 6H), 2.66 (t, J = 7.6 Hz, 2H), 2.52 (t, J = 7.6 Hz, 2H, merged with DMSO-d_6 peak), 1.97 – 1.81 (m, 4H); ^{13}C NMR (100 MHz, DMSO-d_6): 171.8, 168.7, 161.0, 160.8, 159.0, 158.4, 158.3 (q, J = 35.7 Hz), 158.2, 158.16 , 157.2, 157.0, 146.0, 144.6, 144.3, 141.7, 122.3, 120.7, 118.3, 118.1, 115.7 (q, J = 290.2 Hz), 115.3, 54.7, 48.6, 42.3, 35.9, 34.2, 24.5, 24.4, 24.3; peaks for trifluoroacetate salt were observed at 158.3 (q, J = 35.7 Hz), 115.7 (q, J = 290.2 Hz) in ^{13}C NMR; HRMS (ESI) calcd for $C_{29}H_{35}N_{14}O_5S_4$ $[M+H]^+$: 787.1792; Found: 787.1798.

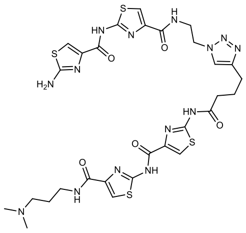

3.0 NMR spectra of compounds

^{1}H and ^{13}C NMR of compound S3:

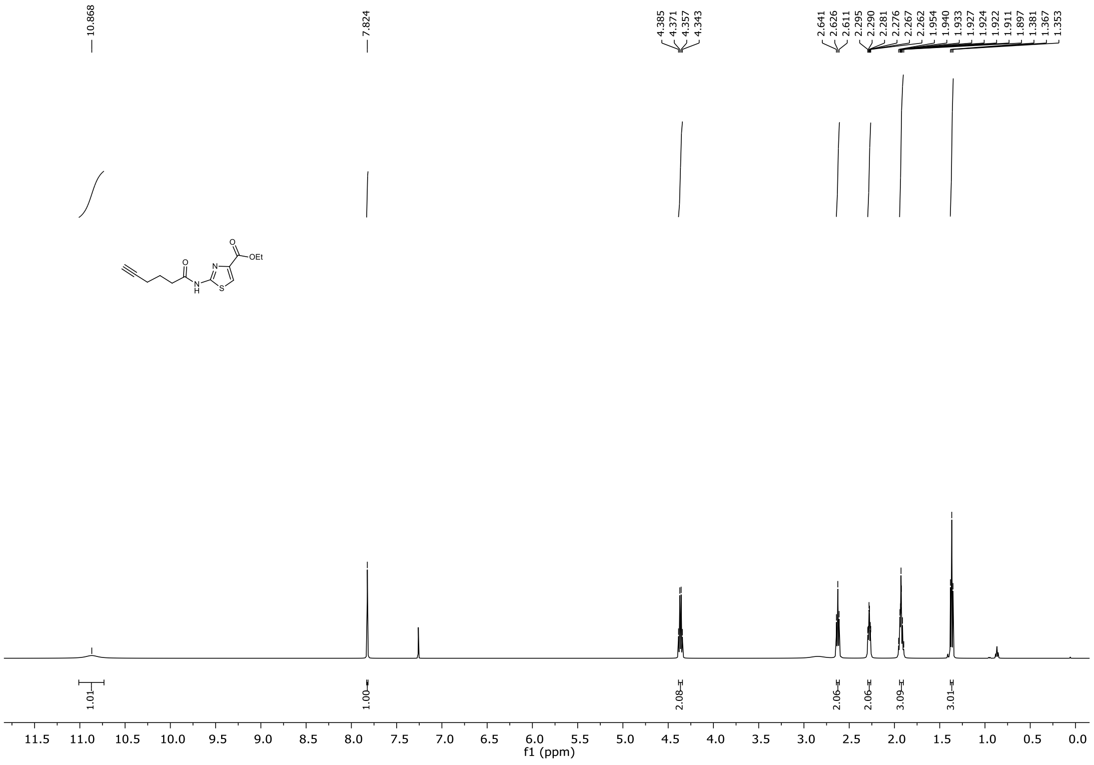

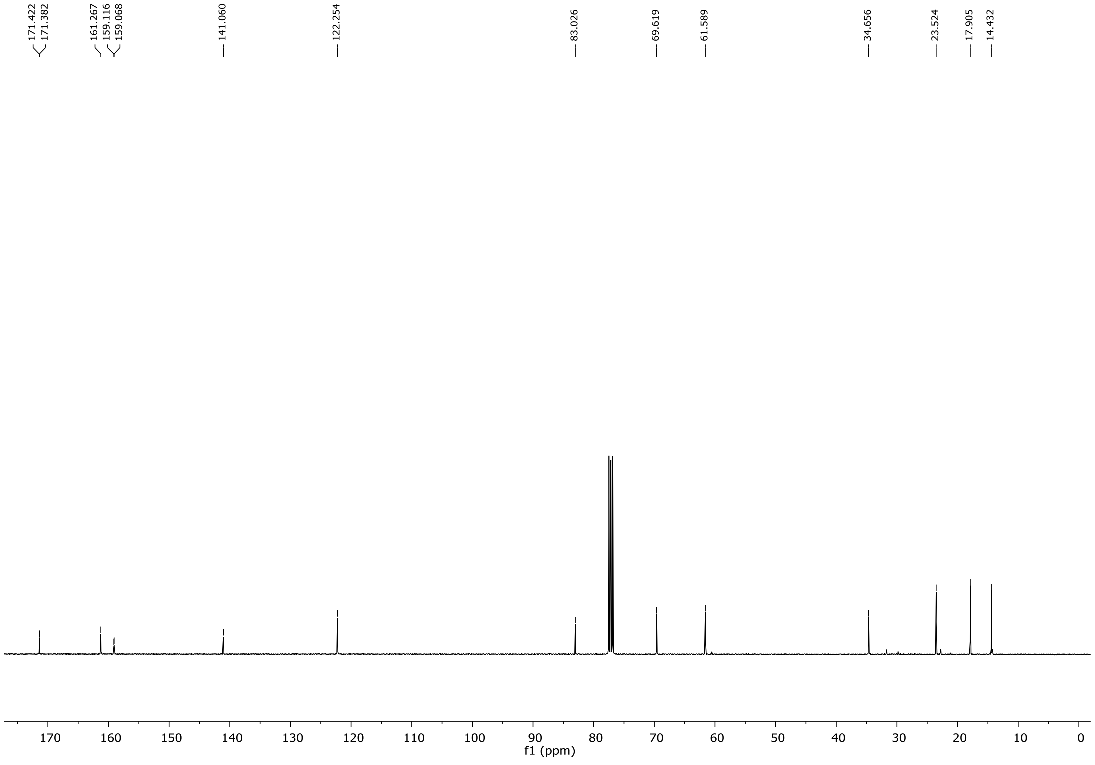

^{1}H and ^{13}C NMR of compound S4:

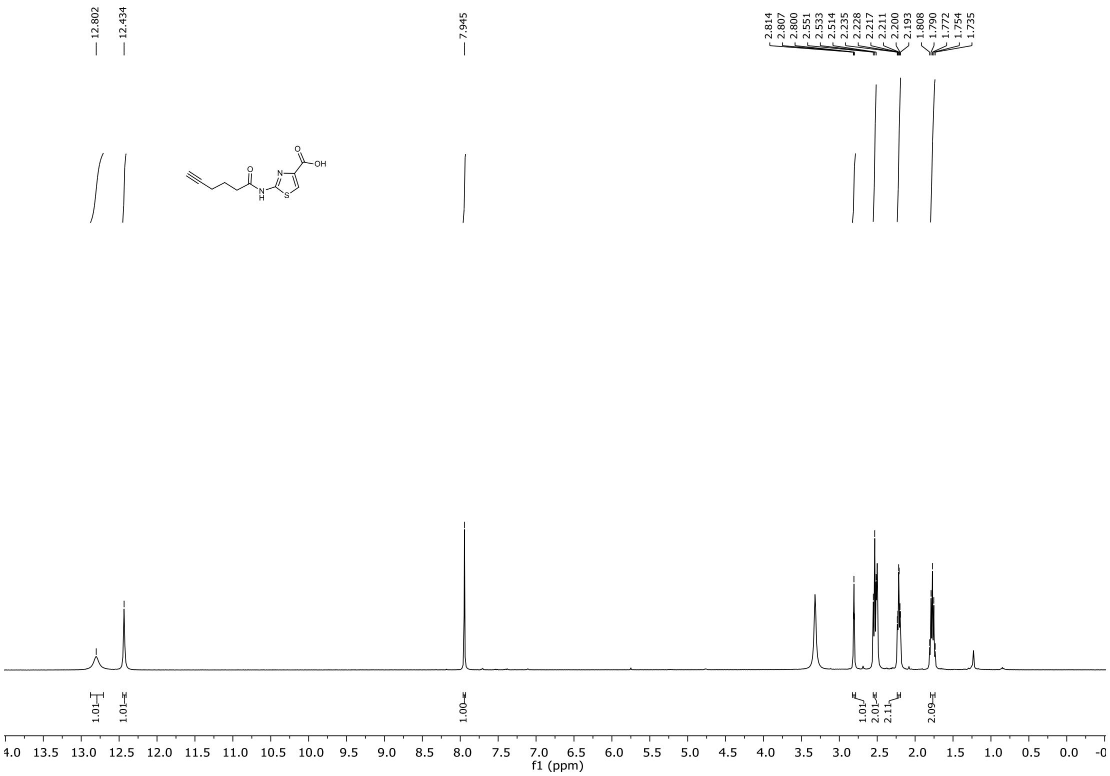

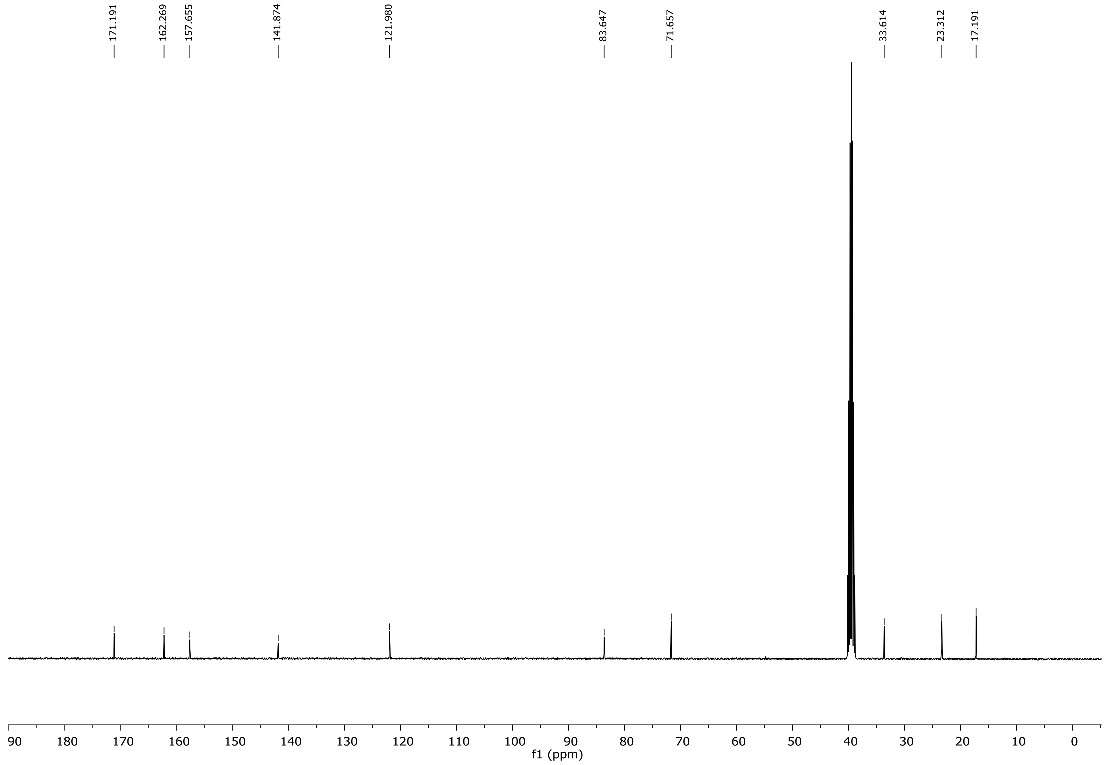

^{1}H and ^{13}C NMR of compound S5:

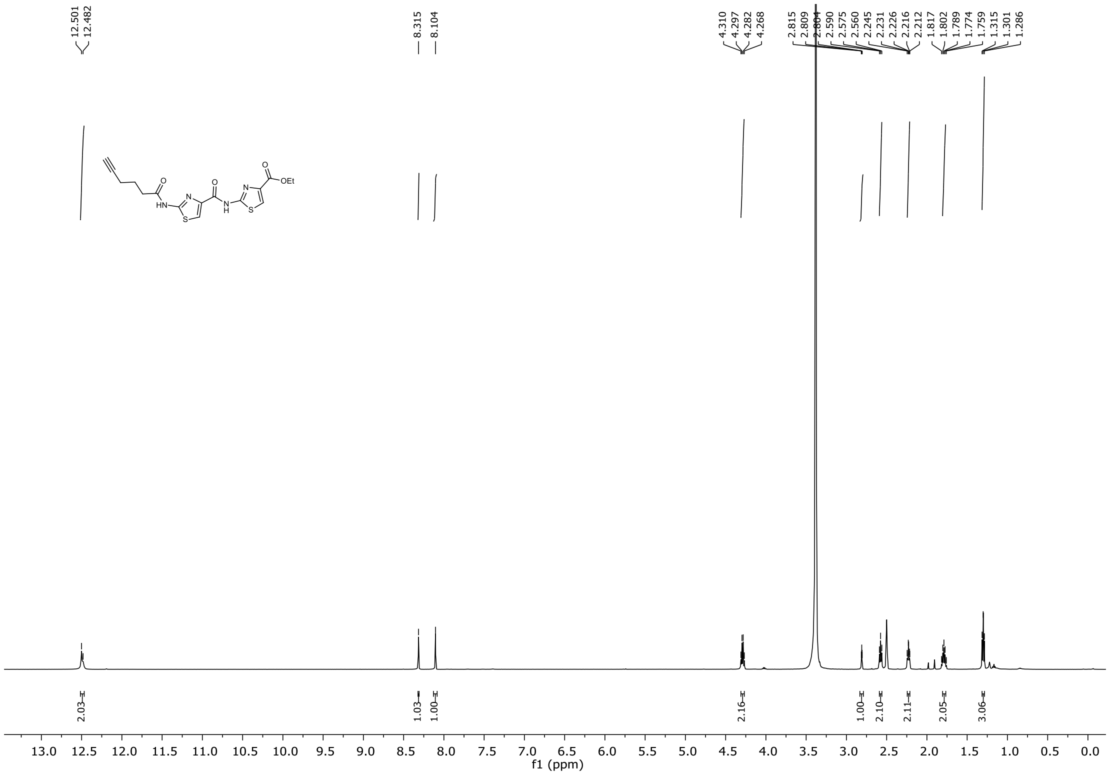

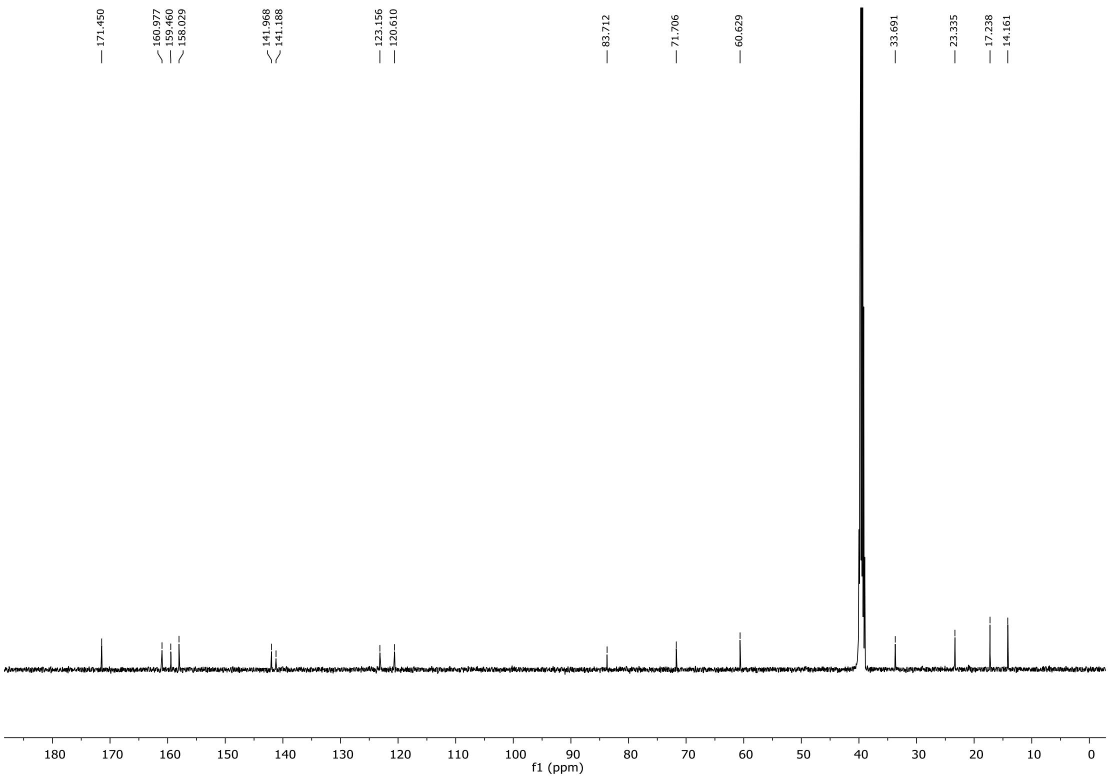

^{1}H and ^{13}C NMR of compound S6:

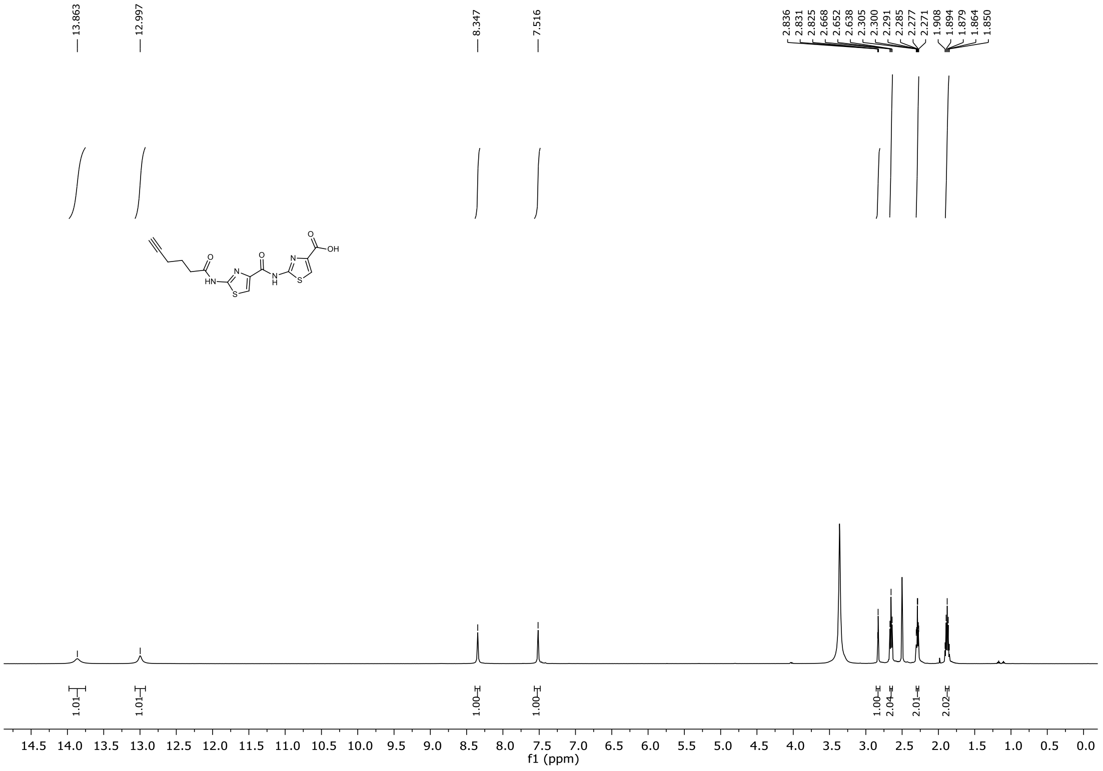

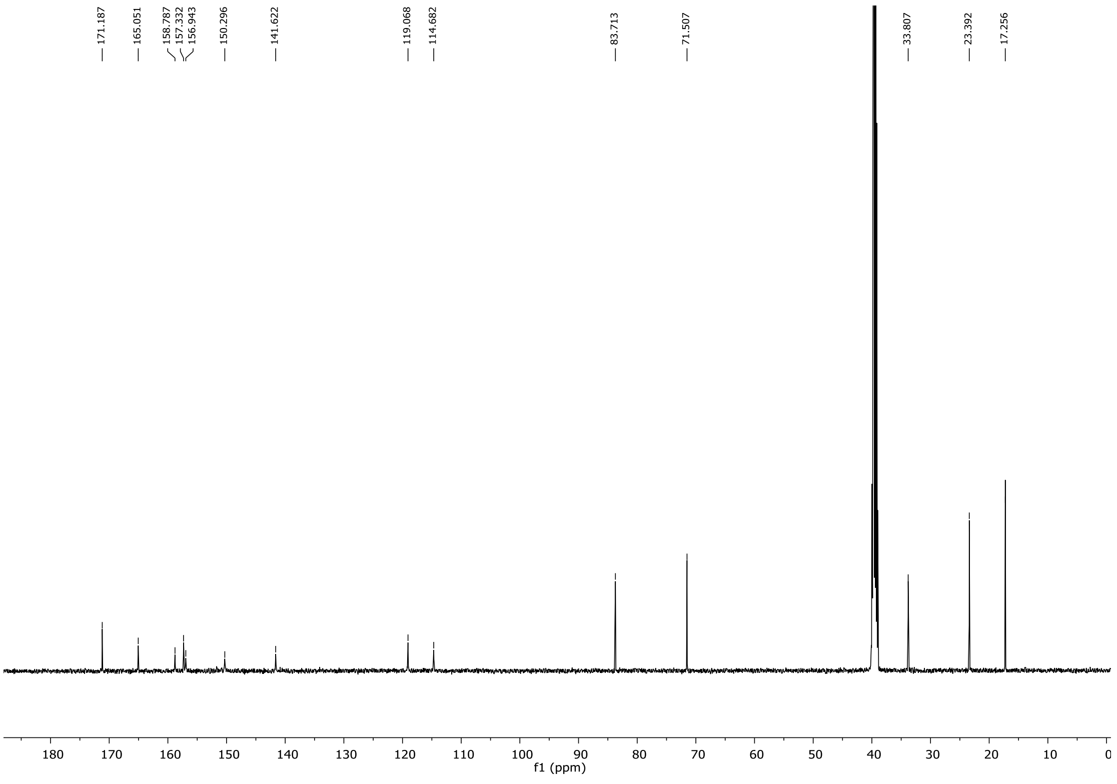

^{1}H and ^{13}C NMR of compound 2:

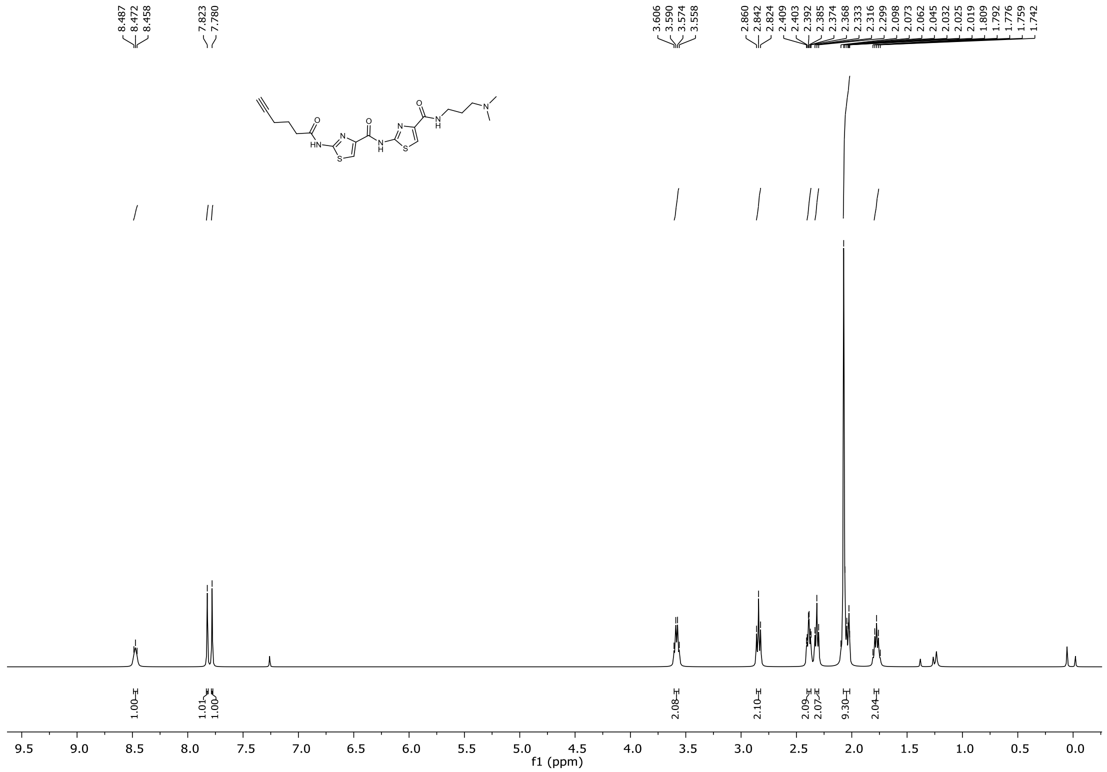

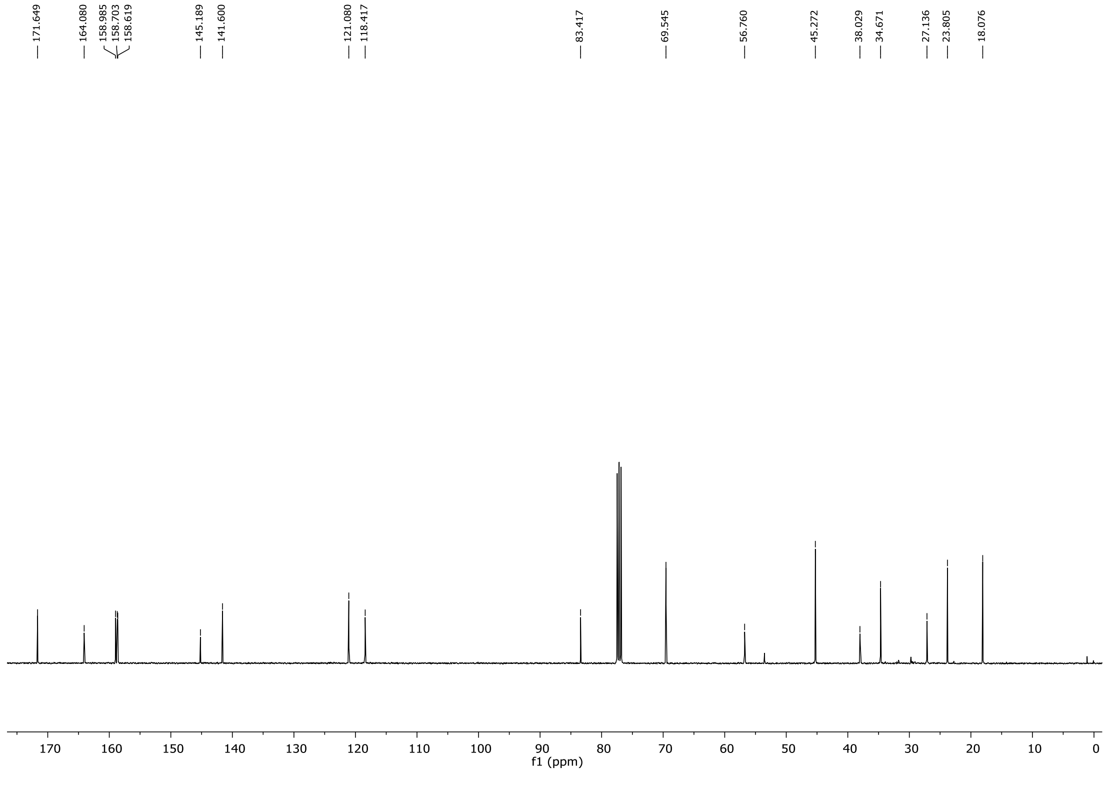

^{1}H and ^{13}C NMR of compound 1:

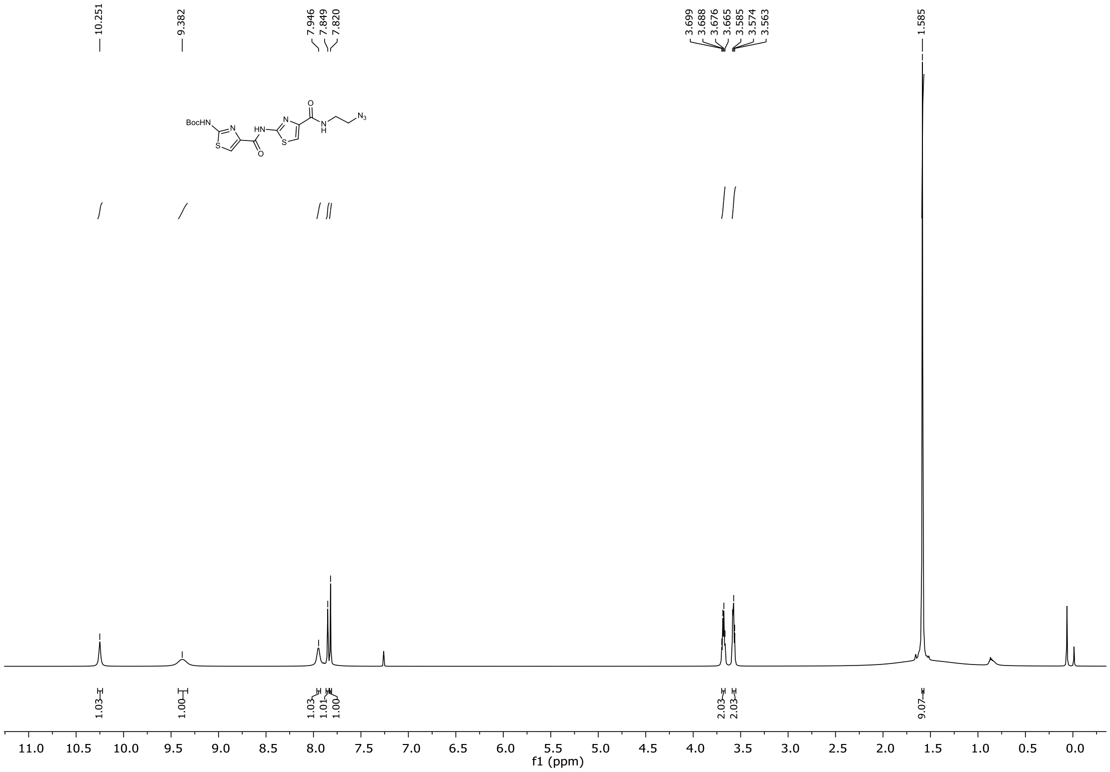

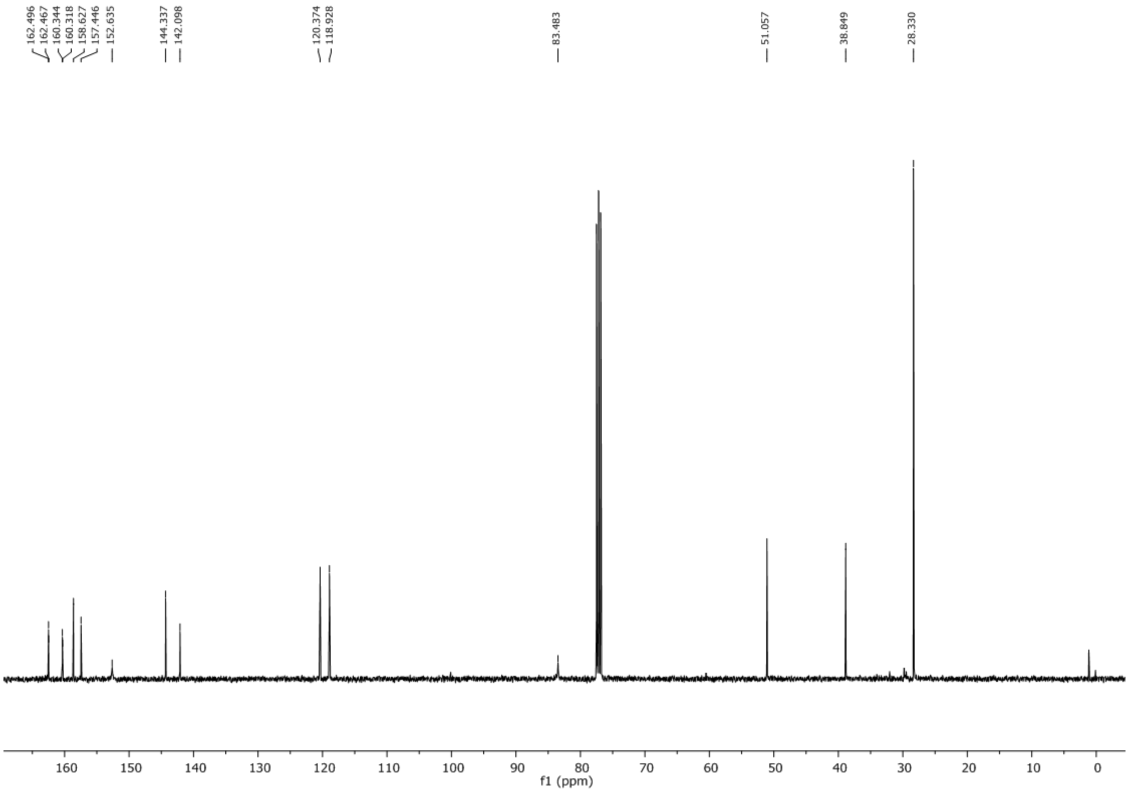

^{1}H and ^{13}C NMR of compound TBP1:

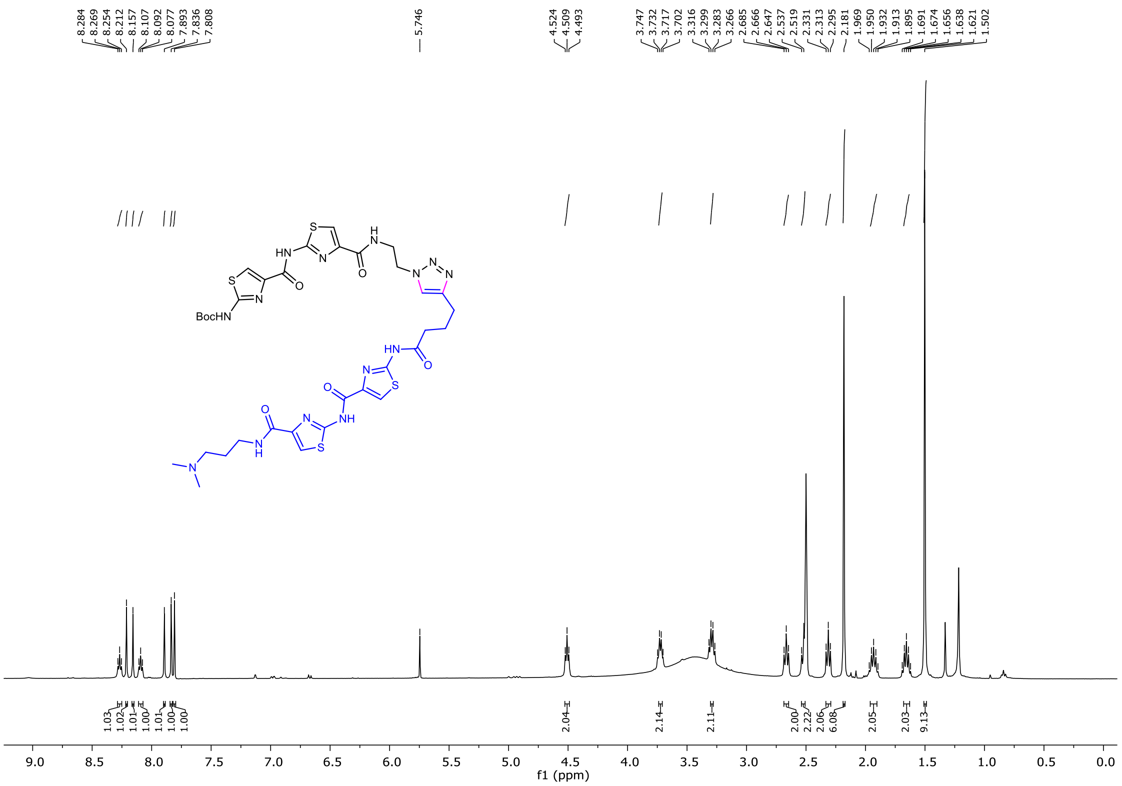

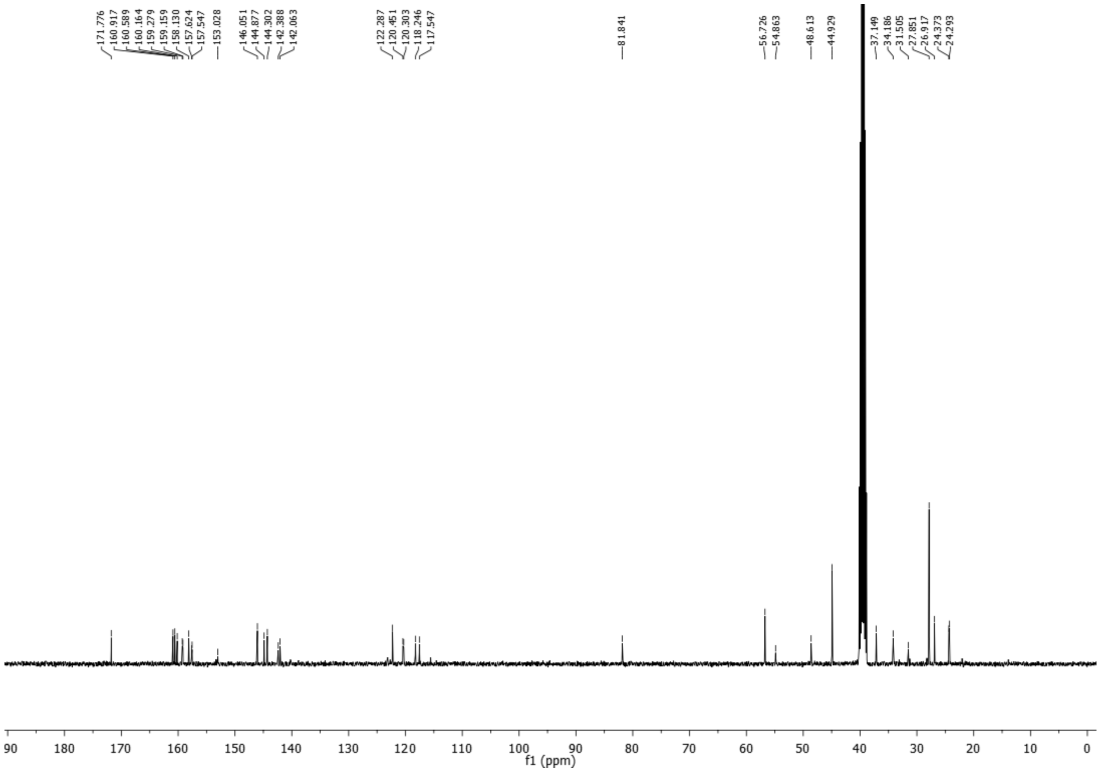

^{1}H and ^{13}C NMR of compound TBP2:

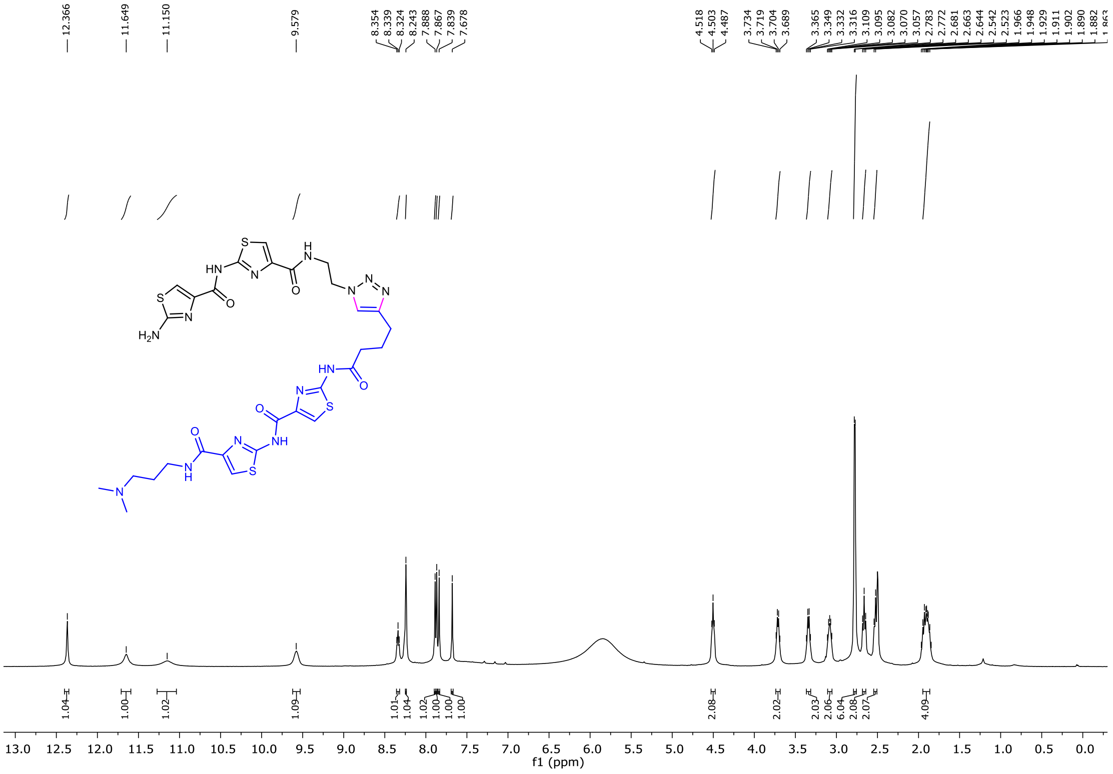

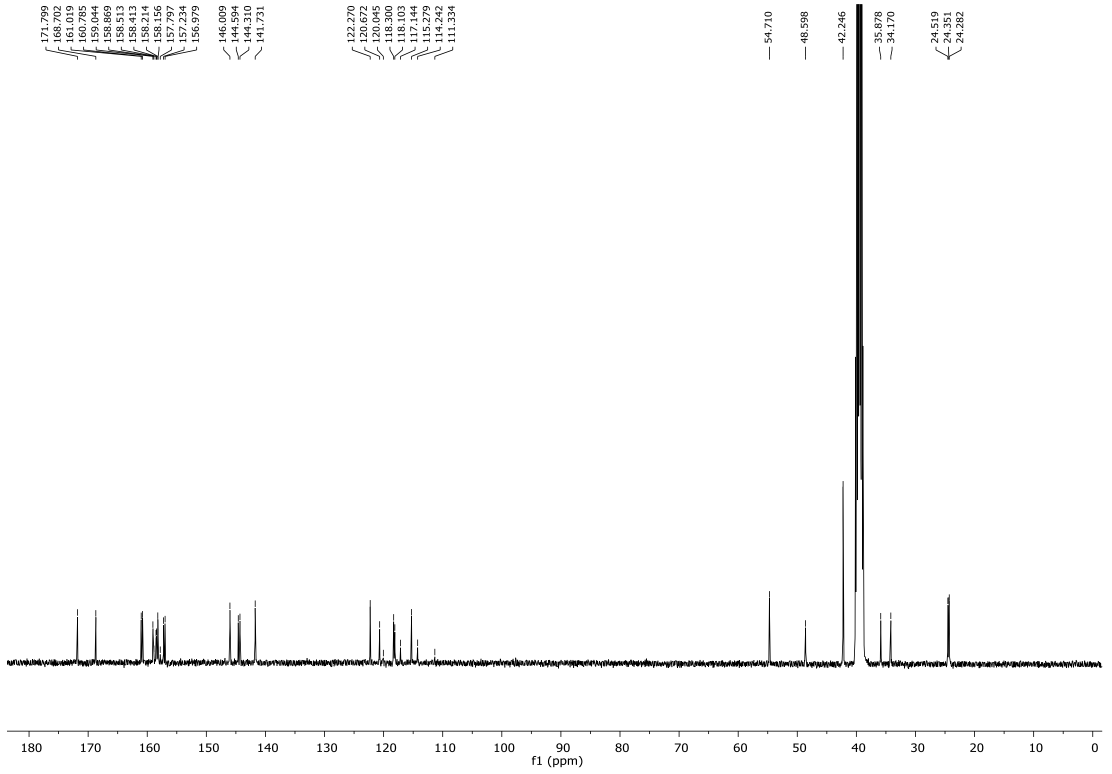

^{13}C-DEPT NMR of compound TBP2:

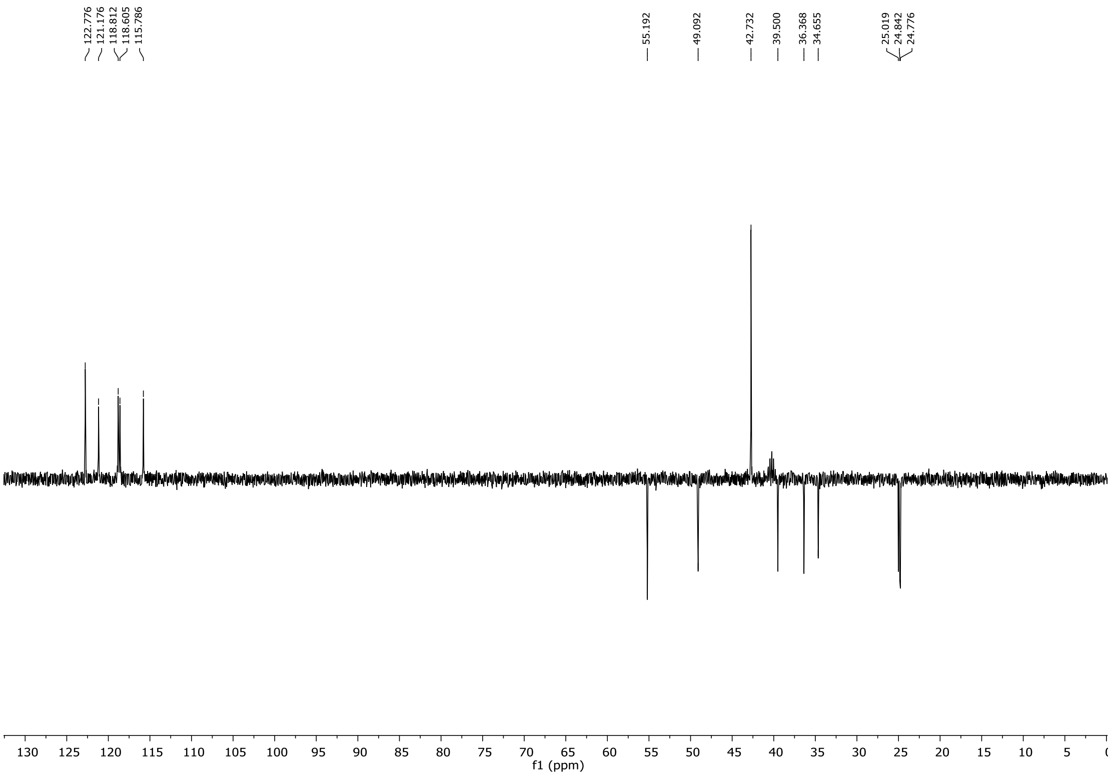

Mass spectrometry of TBP2

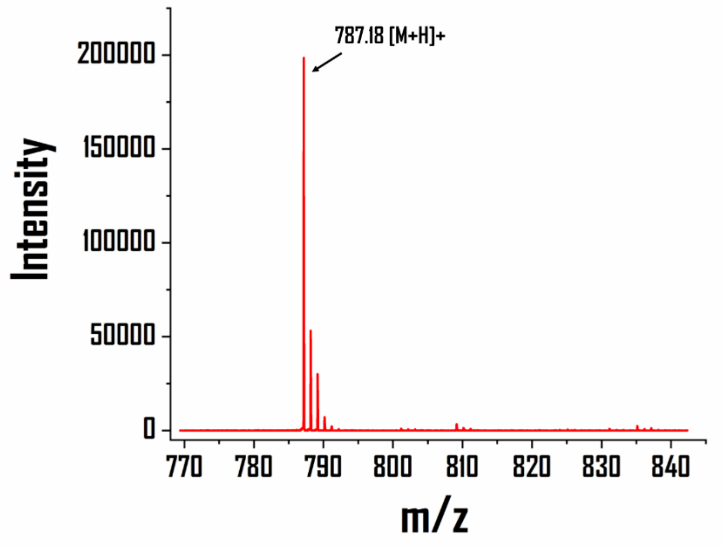

4.0 HPLC analysis

TBP2 was purified by HPLC with ODS-2 C18 column (250 × 4.6 mm, 5 μm) at 25 °C. The compound mixture was prepared with CH_3CN/H_2O (90:10). The mobile phase was taken CH_3CN/H_2O (90:10) with run time of 10-15 min at the injection volume of 10 μL. The flow rate was maintained at 0.5 mL/min. The UV detection was measured at 290 nm.

HPLC chromatogram of **TBP2**

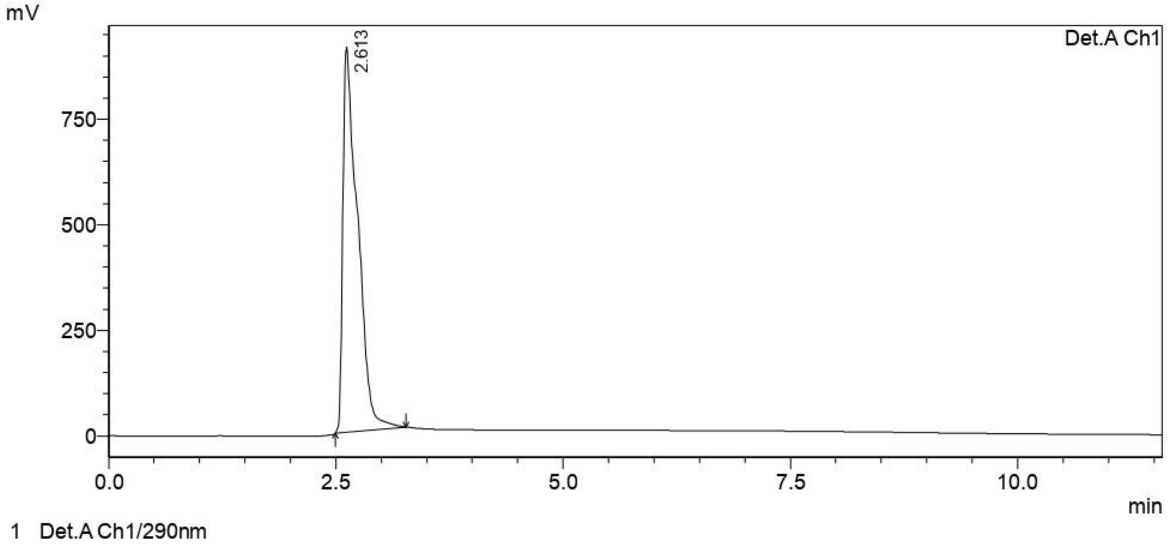

PeakTable

Detector A Ch1 290nm

Peak#	Ret. Time	Area	Height	Area %	Height %
1	2.613	10335829	911516	100.000	100.000
Total		10335829	911516	100.000	100.000

5.0 Lipids, oligonucleotides and chemicals

The common chemicals, lipids, Sephadex G-25 column and unlabeled DNA sequences were procured from Sigma-Aldrich, Bio-Rad and Integrated DNA Technologies (IDT, USA). HPLC purified unlabeled DNA sequences were procured to obtain best results. The reagents used in cell culture were bought from Thermo Fisher Scientific (USA).

6.0 TEM imaging

TEM experiments were carried out in bright-field mode on a JEOL 1200 EX electron microscope, operated at an acceleration voltage of 120 keV. **TBP1** or **TBP2** were diluted in HEPES NaCl or HEPES KCl buffer (pH 7.4) at the con. of 20 μM. The sample was prepared by placing a drop (5 μL) of aqueous dispersions of **TBP1** and **TBP2** on a carbon-coated copper grid and air-dried at room temperature overnight. In case of **TBP2**, multiple vesicular structures can be seen along with the nanofibres as shown in Fig. 1A.

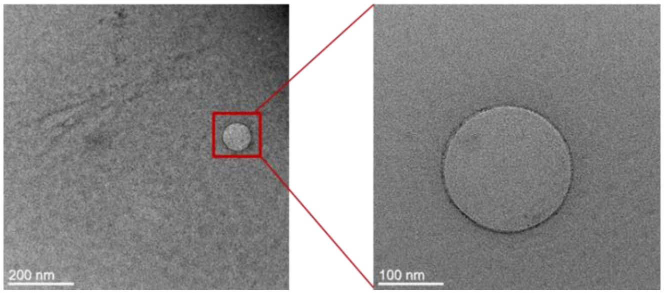

Fig. S2: TEM images of **TBP1** in NaCl buffer (pH 7.4).

7.0 Ion transport study by fluorescence spectroscopy

Preparation of large unilamellar vesicles (LUVs)

Large unilamellar vesicles (LUVs) were formed using a 200 μL 9:1 mixture of 10 mM EYPC (L-α-Phosphatidylcholine egg yolk) and cholesterol in chloroform. After solvent removal and vacuum drying, the resulting thin film was hydrated with 500 μL of buffer (10 mM HEPES, 100 mM NaCl or KCl, pH 6.4) containing 100 μM HPTS (8-hydroxypyrene-1,3,6- trisulfonic acid trisodium salt). Next, the suspension was subjected to six freeze–thaw cycles (liquid nitrogen/water at room temperature) during hydration. The resulting white suspension was then extruded 19 times through a 100 nm polycarbonate membrane to obtain large unilamellar vesicles (LUVs) with an average diameter of ~ 60 nm (as measured by DLS method). The LUVs suspension was separated from extravesicular HPTS dye by using size-exclusion chromatography (Econo-Pac 10DG column, Bio-rad; mobile phase: 10 mM HEPES, 100 mM NaCl or KCl, pH 6.4) and diluted with mobile phase for the desired working concentration.

Monitoring ion transport by HPTS assay[1]

TBP1 or **TBP2** (0-20 μM) was added to a EYPC· LUVMs⊃ HPTS suspension ([EYPC] = 10 mM, [HPTS] = 100 μM) in 10 mM HEPES buffer containing 100 mM MCl [M = Na^+, K^+, Cs^+, Rb^+, Li^+] (490 μL, pH 6.4) followed by subsequent addition of an aqueous solution of NaOH (0.5 M, 5 μL, ΔpH = 1) in a clean and dry fluorescence cuvette. Fluorescence intensity of HPTS at 510 nm upon excitation with 460 nm-light was monitored as a function of time until the addition of 1.0 wt% Triton X-100 (40 μL) at 450 s. Relative fluorescence intensity of HPTS was evaluated by the equation of

$$I = \frac{I_t - I_0}{I_{Lysed} - I_0}$$

Where I_0, and I_t represent the initial, final and I_{Lysed} represent the fluorescence intensities before addition of NaOH, after addition of NaOH and 1 wt% Triton X as lysis buffer, respectively.

The Hill equation was used to determine the EC_{50} values (concentration of molecules to achieve 50% ion transport activity) of the peptidomimetics:

$$I = \frac{1}{1 + (\frac{EC_{50}}{[Channel]n})}$$

I = relative fluorescence intensity, [Channel] = concentration of **TBP1** or **TBP2**, n = Hill co-efficient

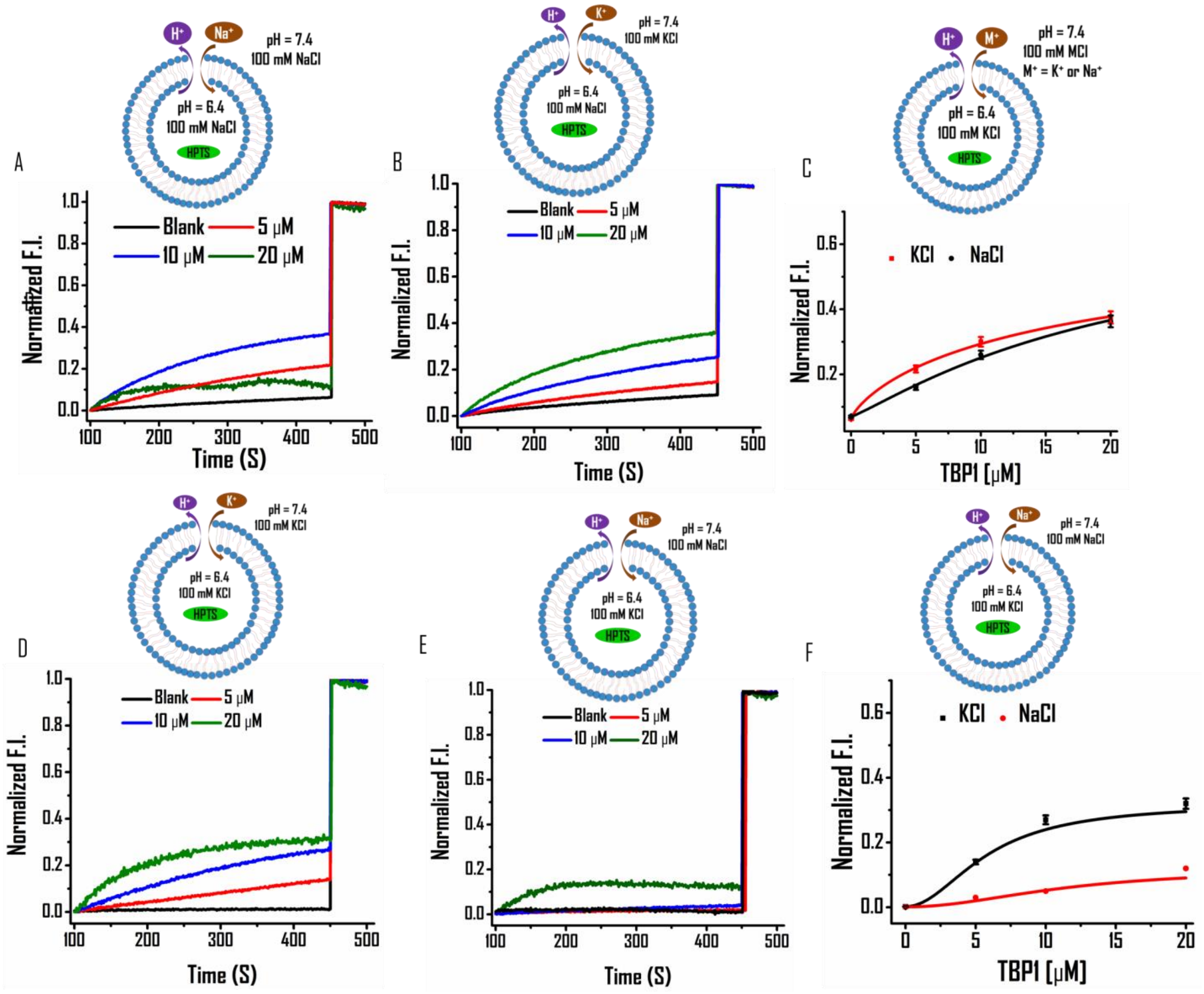

Fig. S3.1: HPTS assay & determination of EC_{50} values for measuring the ion transport activity of **TBP1** in the presence of Na^+ and K^+. Buffer composition: Internal – **(A, B)** 10 mM HEPES 100 mM NaCl (pH 6.4), **(D, E)** 10 mM HEPES 100 mM KCl (pH 6.4), External – **(A, E)** 10 mM HEPES 100 mM NaCl (pH 7.4), **(B, D)** 10 mM HEPES 100 mM KCl (pH 7.4). **(C)** EC_{50} value determination of **TBP1** in the presence of external buffer – 10 mM HEPES 100 mM NaCl or KCl (pH 7.4), internal buffer – 10 mM HEPES 100 mM NaCl (pH 6.4). Data are presented as means ± SD ($n = 3$). **(F)** EC_{50} value determination of **TBP1** in the presence of external buffer – 10 mM HEPES 100 mM NaCl or KCl (pH 7.4), internal buffer – 10 mM HEPES 100 mM KCl (pH 6.4). Data are presented as means ± SD ($n = 3$).

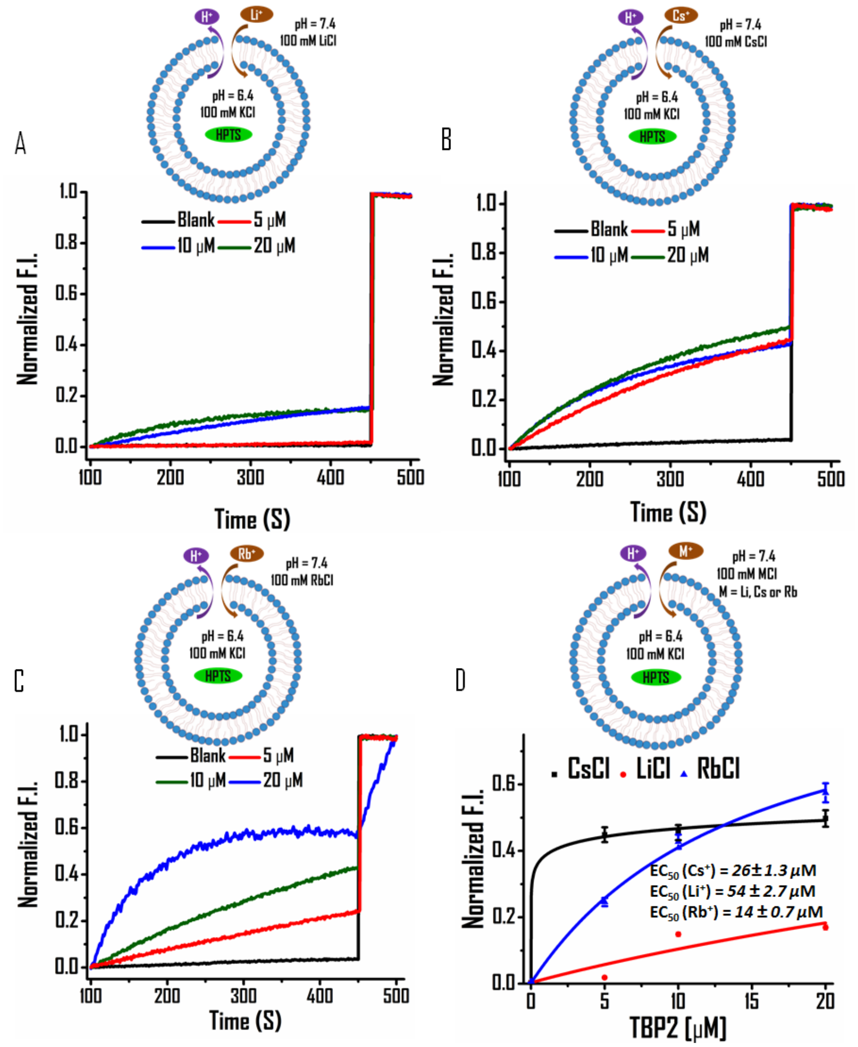

Fig. S3.2: **TBP2** gated ion transport via model lipid bilayer. (A-C) HPTS assay for measuring the ion transport activity of **TBP2** in the presence of Cs^+, Li^+ and Rb^+ ions. Buffer composition: Internal –10 mM HEPES 100 mM NaCl (pH 6.4), External –10 mM HEPES 100 mM CsCl, LiCl or RbCl (pH 7.4). (D) EC_{50} value determination of **TBP2** in the presence of external buffer – 10 mM HEPES 100 mM CsCl, LiCl or RbCl (pH 7.4), internal buffer – 10 mM HEPES 100 mM NaCl (pH 6.4). Data are presented as means ± SD ($n = 3$).

Cl^- transport by lucigenin assay

To determine the **TBP2** mediated transport of chloride ions (Cl^-), fluorescence based lucigenin assay was performed as follows: 1 mM lucigenin with 225 mM $NaNO_3$ filled LUVs were prepared as previously described; and resuspended in 225 mM $NaNO_3$ solution. The transport activity was examined by monitoring the intravesicular lucigenin fluorescence intensity at an emission of 535 nm (λ_{ex} 455 nm).[3] 1 M NaCl was added at 50 s to generate $Cl^-/NaNO_3$ gradient followed by the addition of **TBP2** at t = 200 s. Concentration-dependent quenching of lucigenin fluorescence confirms the Cl^- transport across lipid membrane by ion channel forming compound.

Preparation of carboxyfluorescein (CF) encapsulated vesicles

Liposomes were prepared by using the extrusion procedure. Lipid films were prepared from a mixture of 10 μL of 10 mM egg yolk L-α-phosphatidylcholine (EYPC) and cholesterol (9:1) in chloroform by drying in vacuum for ~ 16 hrs. The film was hydrated with carboxyfluorescein (0.5 mL, 40 mM) in HEPES buffer (10 mM HEPES 100 mM KCl/NaCl pH 7.4). The mixture was then resuspended to form vesicles followed by vortexing. The unilamellar vesicles were extruded through a polycarbonate membrane with a pore diameter of ~1 μm. Free dye was removed by column chromatography using a Sephadex G25 column.

CF release experiments[2]

The carboxyfluorescein release assay was carried out via the formation of CF encapsulated LUVs. CF was encapsulated in large unilamellar vesicles (LUV) prepared from EYPC/cholesterol (9:1) under self-quenching conditions (0.5 mL, 40 mM carboxyfluorescein). **TBP1** or **TBP2** (20 μM) in DMSO was added and mixed with the LUVs in HEPES NaCl buffer (final volume 500 μL). The time resolved fluorescence intensity (Horiba Jobin Yvon Fluoromax

3 instrument) was monitored at 25 °C at 520 nm (λ_{ex} = 480 nm). Total dye release was determined by disruption of the LUVs by the addition of Triton X-100 (40 mM). The percentage of CF release caused by ligands was determined by using the following equation:

$$Leakage\ (\%) = (F_t - F_0)(F_{100} - F_0) \times 100$$

Where, F_t is the time dependent fluorescence intensity and F_0 and F_{100} are fluorescence intensities observed in the absence of **TBP1** and **TBP2** and after Triton X-100 treatment, respectively.

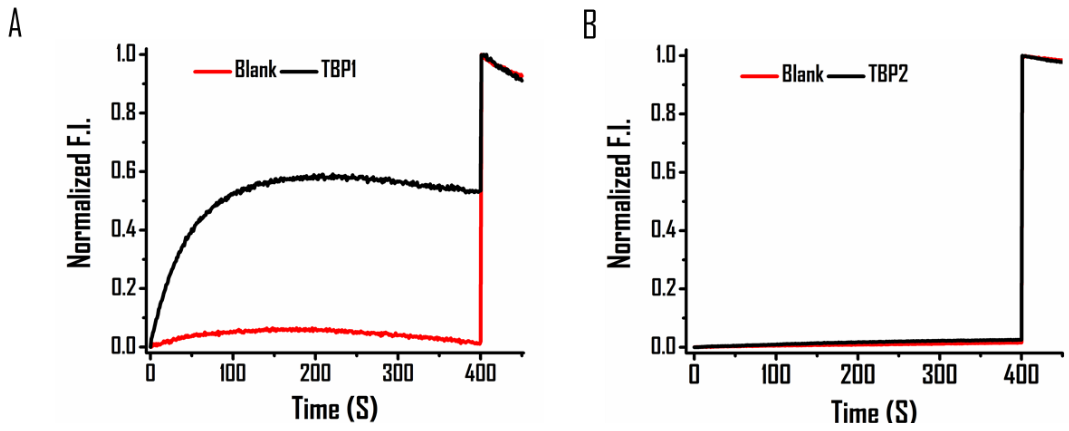

Fig. S4: Carboxyfluorescein (CF) release assay in the presence of **(A) TBP1** and **(B) TBP2** (External buffer: 10 mM HEPES 100 mM NaCl, pH 7.4, Internal buffer: 10 mM HEPES 100 mM NaCl, pH 7.4).

8.0 Formation of giant unilamellar vesicles (GUVs)

GUVs were prepared by electroformation technique (Vesi Prep Pro, Nanion, Germany). 10 μL of a 10 mM solution of EYPC and cholesterol (9:1) in chloroform was spread evenly on the indium tin oxide (ITO) coated glass slides within the “O” ring area. The solvent was evaporated at room temperature and the slides were dried overnight under vacuum. Then, ITO slides were assembled in the Vesi Prep Pro and filled with 275 μL of sorbitol solution (1 M). A sinusoidal AC field of 3 V and 5 Hz was applied for 2 h at 25 °C temperature. The prepared GUV solution was collected and subjected to patch-clamp experiments.

9.0 Confocal imaging in GUVs and cells

GUVs were suspended in 10 mM HEPES, 100 mM KCl or 100 mM NaCl (pH 7.4) buffer and incubated with **TBP1** or **TBP2** (20 μM) and nile red (Sigma, USA) for 5 minutes. After incubation, the mixture was placed on the fluodish and observed under Confocal Microscope (Zeiss, Germany). For control slides, GUVs were incubated with either **TBP2** or Nile red. At least 4 fields per slide and three independent sets were examined. HeLa cells were treated with **TBP1** or **TBP2** (8 μM) following nile red (10 μM) incubation 10 minutes prior to imaging. The images obtained were processed using ImageJ software.

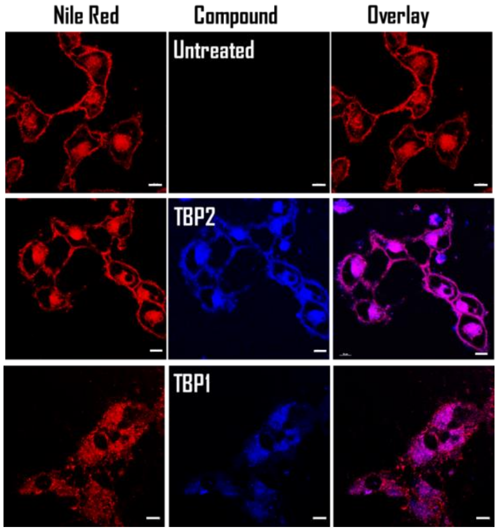

Fig. S5: Membrane colocalization of **TBP1** and **TBP2** with nile red in HeLa cells after 10 minutes of incubation. Scale bars represent 10 $\mu\mu$m.

10.0 Safranin O assay

We have performed Safranin O assay to investigate **TBP2** dependent membrane polarization using unilamellar vesicles. For membrane polarization experiments, GUVs were prepared using 9:1 mixture of 10 mM EYPC and cholesterol via electroformation technique. In a fluorescence cuvette, 25 μL of GUV solution was suspended in 475 μL 10 mM HEPES, pH 6.4 containing the corresponding salt 100 mM NaCl or KCl. Next, safranin O was added at a final concentration of 1 μM. The fluorescence (with or without **TBP2**) was monitored for 600 sec at an excitation of 522 nm and emission of 581 nm. The safranin O data suggest that **TBP2** alters lipid membrane polarization in the presence of NaCl (external) and KCl (internal) buffer solution.

11.0 Conductance measurements by automated patch-clamp technique

Conductance measurements were carried out using the Port-a-Patch setup (Nanion, Munich, Germany). First, a borosilicate glass chip (NPC chip, Nanion, Germany) with 3-5 mΩ was loaded with symmetrical working buffer containing 10 mM HEPES 1 M MCl [M = Na^+, K^+, Cs^+, Li^+, Rb^+] buffers (pH 7.0) in both *cis* and *trans* compartments and Ag/AgCl electrodes were placed on both sides of the NPC chip. Next, bilayer membrane with >1 Giga Ohm resistance was constructed across the micrometer-sized aperture in the NPC chip by adding GUV suspension and applying a mild negative pressure (-10 mbar). **TBP2** (20 μM) was added to the *cis*-side of the chamber. Current traces were recorded using an HEKA EPC 10 patch clamp amplifier with a built-in 1 kHz 4 pole Bessel low-pass filter and a Digidata 1322A digitizer. I-V curve was generated using a voltage ramp from -80 mV to +80 mV. Data analysis was performed using Clampfit 10.2 software.

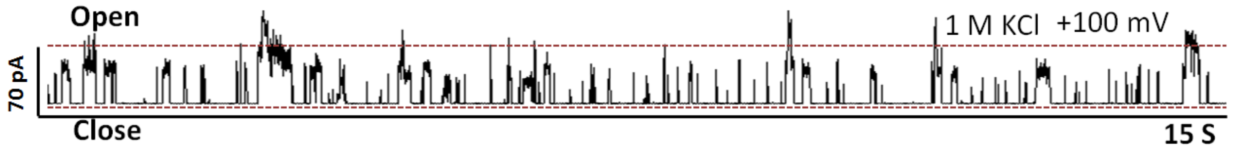

Fig. S6: Current measurement of **TBP2** gated ion channel in the presence of 1 M KCl at +100 mV.

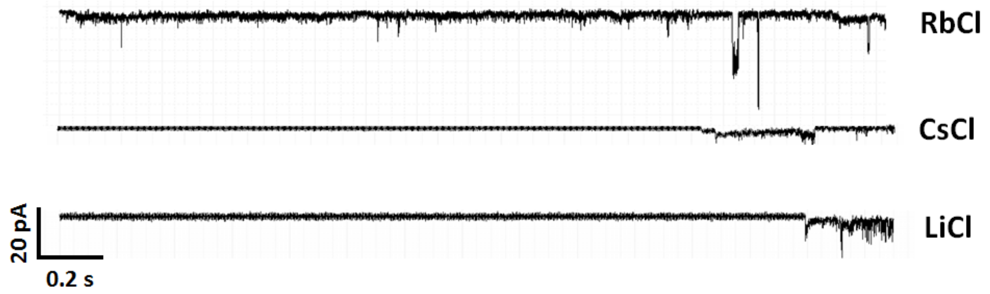

Fig. S7: Current measurement of **TBP2** gated ion channel in the presence of 1 M RbCl, CsCl or LiCl at -80 mV.

12.0 Dynamic light scattering (DLS)

The average hydrodynamic radius (Rh) of **TBP2** embedded in vesicles was determined by DLS using a particle size analyzer (Zetasizer Nano-ZS, Malvern Instruments Ltd.). The sample was

prepared by gel filtration of embedded LUVs suspension to a final concentration of 10 μM **TBP2** in 40 μl of LUV suspension in 10 mM HEPES, 100 mM NaCl, pH 7.4. In addition, the hydrodynamic radius and the relative abundance of **TBP2** formed vesicles (separated by centrifugation method) were measured in HEPES-NaCl buffer. Measurements were recorded at room temperature (25°C) with a scattering angle of 173°. Experiments were carried out at least in triplicate. The statistical analysis was accomplished with student's 't' test (±SD).

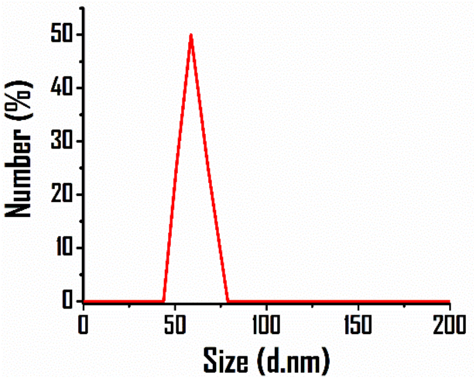

Fig. S8: Hydrodynamic radius of LUVs in HEPES NaCl buffer (pH 7.4)

13.0 Computational details

Quantum chemical calculations

The **TBP2** molecule was constructed using the GaussView visualizer and optimized using Density Functional Theory (DFT), employing both of the B3LYP/6-31G(d,p), and M06-2x/6-31G(d,p) level of theories implemented in the Gaussian09 package.[3-6] The M06-2x/6-31G(d,p) level of theory was used since it has the capability of better estimating the intra- and intermolecular non-covalent interactions, which are crucial for stabilizing the folded form of **TBP2**. To computationally mimic the ion transport through the nanopore created by the assembled **TBP2** molecules on top of each other, we first optimized the geometry of a **TBP2** dimer using the M06-2x/6-31G(d,p) level of theory. The resulting geometry was utilized for the construction of a vertical assembly of six molecules. The number of **TBP2** molecules in the assembly was chosen specifically so as to match the thickness of a lipid bilayer with the height of the assembly. The assembly was then subjected to a multistep molecular dynamics minimization protocol for structural relaxation since DFT could not be used for the optimization of such a large supramolecular architecture.

Molecular dynamics simulations

After molecular dynamics minimization of the vertically arranged **TBP2** molecules creating the ion channel, we inserted the whole supramolecular structure into a pore of similar dimensions created in a POPC lipid bilayer. The lipid membrane of the dimension 10 nm × 8 nm was built in the XY directions using the "Membrane builder" plugin implemented in the VMD package. Our in-house tcl scripts were used for the generation of a pore of the dimension 2.2 nm × 1.2 nm within the lipid membrane by removing lipid molecules. After insertion of the ion channel, we properly solvated the lipid-channel system so that at least 2 nm space was occupied over and below the lipid along the z axis. Required numbers of sodium/potassium and chloride ions were added to two separate systems so that the overall ionic concentrations become 0.15 molar, a necessary requirement for mimicking the physiological environment. Thereafter, we performed a multistep minimization-equilibration procedure to relax and stabilize the systems. The detailed procedure for the mentioned protocol can be found elsewhere.[7-9]

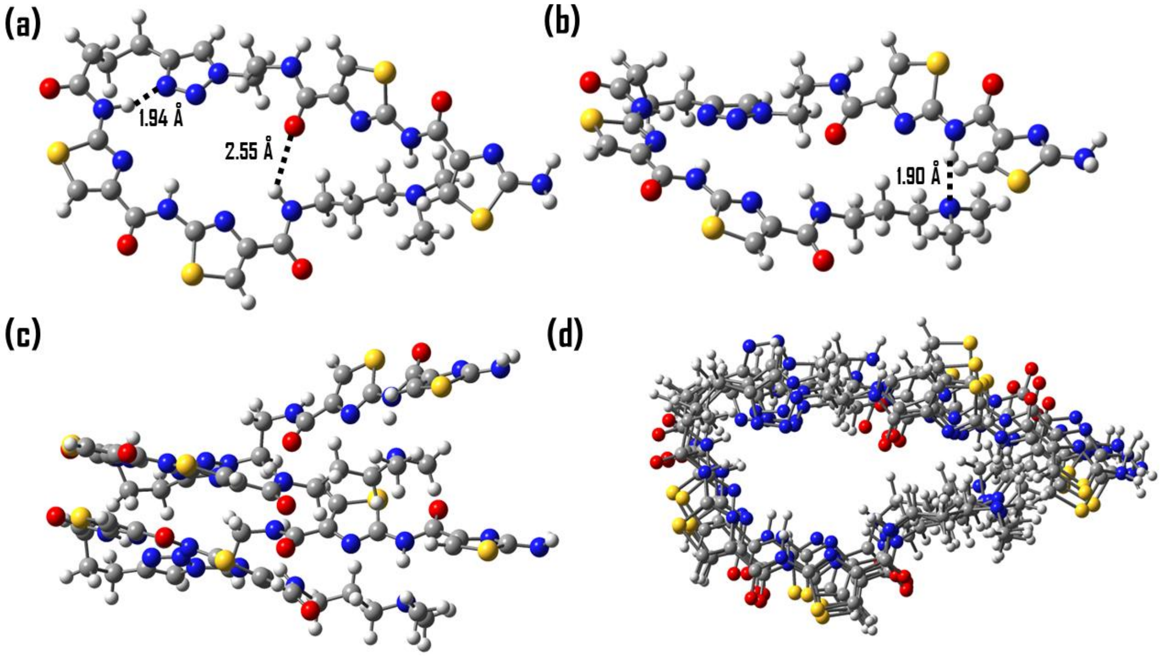

Fig. S9: (a) Top, and (b) side views of the optimized structure of a single **TBP2** molecule at the B3LYP/6-31G(d,p) level of theory. The hydrogen bond distances are indicated in the Fig.s. (c) Optimized structure two vertically placed **TBP2** molecule at the M06-2x/6-31G(d,p) level. (d)

Top view of the vertically stacked arrangement of six **TBP2** molecules showing the ion channel, as obtained from the periodic propagation of an optimized **TBP2** dimer followed by molecular dynamics minimization.

After the equilibration, we opted for different simulation protocols. First, the NaCl and KCl solvated systems were subjected to production simulations in the isothermal-isobaric (NpT) ensemble at 310 K to check whether the cations can enter and pass through the pore spontaneously. On the other hand, we opted for free energy calculations for the translocation of sodium and potassium cations through the ion channel. To this regard, we first moved a cation from the aqueous medium and placed it at one end of the ion channel and then applied a biasing force in accordance with the adaptive biasing force (ABF) module implemented in NAMD 2.12, so that the cation can enter the ion channel and pass through it.[10] The force induced movement of the cation was continued until the ion is released through the other end of the ion channel. The whole process allows us to calculate the minimum free energy required to pass the ion from one side of the lipid membrane to the other side and is represented in terms of potential of mean force (PMF). To calculate the PMF's for single cations, the center of mass (COM) distance (d) between the ion channel and the cation was chosen as the reaction coordinates which were divided into several overlapping windows of 0.1 nm widths. Each of these windows was further divided into small bins of 0.02 nm widths. A harmonic force of 50 kcal/mol/$Å^2$ was applied to the upper and lower boundaries of the reaction coordinates and 20 ns production ABF simulations were performed for each window at 310 K. The vectorial distance along the positive direction of the z axis with respect to the center of mass of the ion channel is represented with positive values while distances along the negative direction are represented with a negative sign. To reduce sampling issues and avoid memory effects, we performed each PMF calculations at least two times and the averages are reported. Free energy of the cations completely released from the ion channel is set to zero, where the ion is non-interacting with the ion channel or the lipid membrane. During the PMF calculations, the atoms of the ion channel were harmonically constrained with a mild force of 2 kcal/mol/$Å^2$, and the entire lipid and other atoms were set free. All ions were allowed to move freely and biasing force was applied only on the cation which was under the application of the adaptive biasing force.

Isothermal conditions in all of the simulations were maintained using the Langevin dynamics with a damping coefficient of 5 ps^{-1} and the Langevin piston method was used to

maintain 1 atm pressure.[11-13] A 100 fs piston period, 50 fs damping time constant and 300 K piston temperature was employed to introduce the constant pressure coupling. The Particle mesh Ewald (PME) method with 1 Å grid was employed to calculate the periodic electrostatic interactions and a 2 fs time step was used to integrate the classical equations of motions using the Velocity Verlet algorithm. Covalent bonds containing hydrogen atoms were made rigid employing the SHAKE algorithm.[14] Non-bonded interactions were calculated with a cut-off distance of 12 Å and the atomic coordinates were stored after every 20 ps for trajectory analysis. All the initial configurations were prepared using the Packmol code. The NAMD 2.12 package was used for classical molecular dynamics simulations, VMD for visualization and in-house Tcl scripts for the analysis of the simulation data.[15-16] To model the molecules involved in the formation of the ion channel, we have used the CGenFF parameters, and TIP3P model was used to model water molecules.[17-19] Further, the charges obtained from the CGenFF library were confirmed from quantum chemical optimization of a single molecule using the MP2/6-31G(d) level of theory and employing the implicit solvation model with water as the solvent, as implemented in the Gaussian09 package.[20-21] The quantum chemical calculation protocol was in accordance with the CGenFF parametrization method, and both the charges were found to be very similar to each other.[17]

To examine the stability of the aggregate, formed by **TBP2** molecules, we performed a 2500 ns (2.5 μs) long simulation with a **TBP2** assembly comprising six molecules placed inside a lipid pore. As observed from Fig. S9(a), the structure of the stacked arrangement of the six **TBP2** molecules remains unchanged throughout the entire trajectory, without the need for any restraining force. Furthermore, from the visual inspection of Fig.s S9(b) and S9(c), it is evident that the ion channel remains intact even after 2.5 μs, with no **TBP2** molecule being expelled from the membrane. The stability of the ion channel was further supported by the average heavy-atom RMSD, which varied between 0.25 and 0.38 nm. Moreover, the average intermolecular distance between **TBP2** molecules did not change appreciably as evidenced by the difference between the maximum and minimum distance being only ~0.15 nm. Minor reorganizations of the **TBP2** assembly occurred due to the structural pressure exerted by the lipid molecules in the immediate vicinity of the pore, as observed by fluctuation in RMSD and intermolecular distance around 1400 ns and 2000 ns. However, despite these fluctuations, **TBP2** channel remained intact, and the final structure closely resembled the initial configuration.

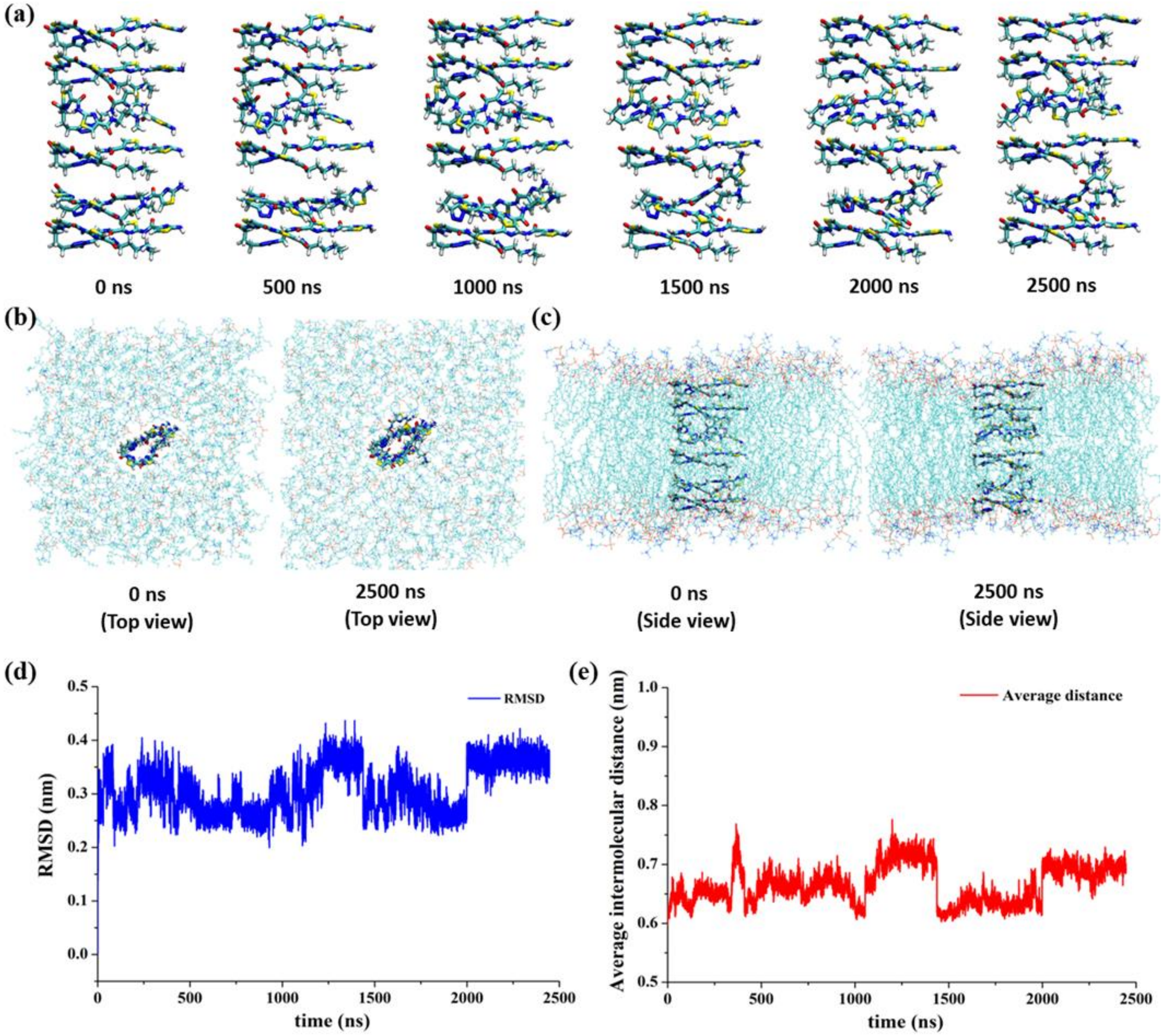

Fig. S10: (a) Snapshots representing structures of the six **TBP2** molecules embedded in the lipid pore at different time instants spanning 2500 ns of production simulation time. (b) Top, and (c) side views of the initial (0 ns) and final (2500 ns) structures of the lipid membrane containing the ion channel. (d) Root mean squared displacement (RMSD) of the heavy atoms for the **TBP2** molecules, and (e) the average distance between consecutive **TBP2** molecules throughout the trajectory.

These findings suggest that severe damage to the **TBP2** assembly and significant changes in the aggregation pattern within the lipid pore can be ruled out even at longer time scales. Additionally, it is apparent that the number of **TBP2** molecules is sufficient to span the entire thickness of the lipid membrane.

14.0 Cell culture

The HeLa (Cervical cancer), A549 (Lung Carcinoma), K562 (Leukemia) and NKE (normal kidney epithelial) cell lines were procured from the National Centre for Cell Science (NCCS, Pune). The cancer cells were grown in DMEM (Gibco, USA) (for HeLa and HEK293T), DMEM:F12K (1:1) (for A549), RPMI 1640 (Gibco, USA) (for K562 and NKE) supplemented with L-glutamine, D-glucose, penicillin–streptomycin (Invitrogen), 10% fetal bovine serum (Gibco, USA). Cells were allowed to grow in six well plates (~ 10^6 cells each well) or 96 well plates and incubated for 24 h at 37 °C with 5% CO_2 before treatment with **TBP2**.

15.0 Measurement of intracellular Na^+ concentration

HeLa or A549 cells were cultured in 96 well plates and incubated with 10 μM fluorescent Na^+ probe [sodium-binding benzofuran isophthalate acetoxymethyl ester (SBFI-AM)] in culture media for 1.5 h at 37 °C. Cells were washed with 1x PBS to remove the free extracellular SBFI-AM following treatment with **TBP2** at different concentrations (0, 1, 2, 5, 8, 20 μM) with 2 h incubation period at 37 °C. The fluorescence of SBFI-AM (λ_{ex} = 350 nm, λ_{em} = 527 nm) was measured in a microplate reader (Molecular Devices, USA).

Measurement of intracellular K^+ concentration

HeLa or A549 cells were cultured in 96 well plates and incubated with 10 μM fluorescent K^+ probe [potassium-binding benzofuran isophthalate acetoxymethyl ester (PBFI-AM)] in culture media for 1.5 h at 37 °C. Cells were washed with 1x PBS to remove the free extracellular PBFI-AM following treatment with **TBP2** at different concentrations (0, 1, 2, 5, 8, 20 μM) with 2 h incubation period at 37 °C. The fluorescence of PBFI-AM (λ_{ex} = 350 nm, λ_{em} = 527 nm) was measured in a microplate reader (Molecular Devices, USA).

16.0 Cell growth inhibition assay

To assess the cytotoxic property of **TBP1** and **TBP2**, we carried out the XTT (2, 3-bis-(2-methoxy-4-nitro-5-sulfophenyl)-2H-tetrazolium-5-carboxanilide) assay in different cancer and normal cell lines. Growth inhibition experiments were performed in triplicate on 96-well plates from lower (sub-micromolar) to higher (submilimolar) doses. Cells were grown (~ 10^5

cells/well) in 100 μl of culture medium and treated with increasing concentration of the compounds (0 − 100 μM) followed by incubation for 24 hours. The XTT/phenazine methosulfate (PMS) reagent was prepared by mixing 1 mg/mL of XTT in 3 mL of culture medium with subsequent addition of 7 μL of 10 mM PMS solution [dissolved in phosphate-buffered saline (PBS)]. This freshly prepared mixture solution (25 μL) was then directly added to each well and incubated for 2 h at 37 °C. The absorbance values of XTT formazan were read at 450 nm on a Multiskan FC microplate spectrophotometer (Thermo Scientific). The calculation of cell growth percentage was done using the below equation:

% of cell viability = (OD of treated cells/OD of untreated control cells) x 100

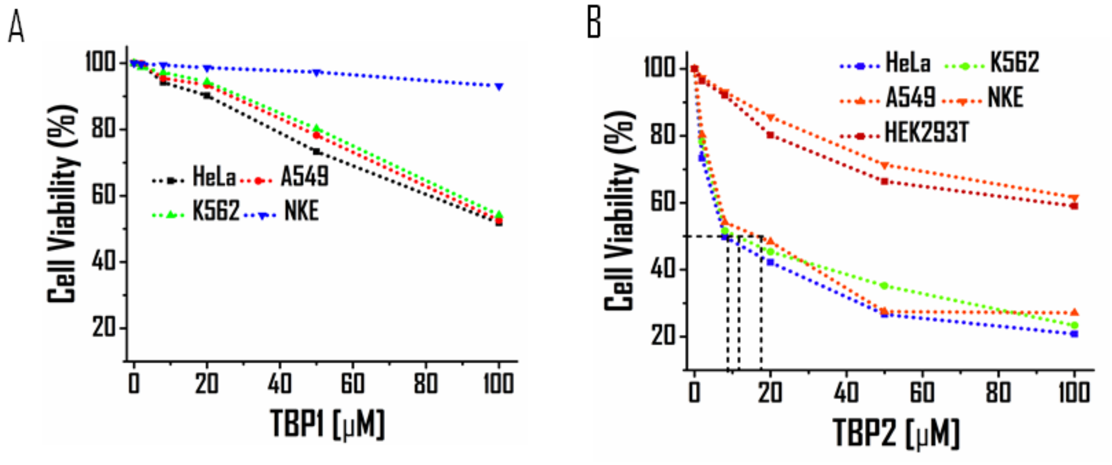

Fig. S11: Cell viability assay for (A) **TBP1** and (B) **TBP2** in different cancer (HeLa, K562, A549) and normal kidney epithelial (NKE) and Human embryonic kidney epithelial (HEK293T) cell lines. Experiments were performed at least three times (n = 3) independently.

17.0 Immunofluorescence via confocal microscopy

Cells cultured on sterile glass cover slips were kept untreated or treated with **TBP1** or **TBP2** (8 μM) for 24 h. Next day, cells were washed few times with 1X PBS and fixed in chilled acetone-methanol (1:1) following permeabilization with 0.03% saponin/PBS solution. After blocking with 3% BSA solution (diluted in 1X PBS), immunofluorescence was performed by incubating the cells with BG4 antibody [(Merck, USA, Catalog no. MABE1126), (1:200 dilution in 1X PBS)] overnight, and Alexafluor 647-conjugated secondary antibody (Invitrogen, Catalog no. A56576) (1:1000 dilution in 1X PBS) for 2 h on the next day. In final step, antified solution

(Invitrogen) was used to mount the cover slips. The BG4 emission (570−670 nm) was taken with the excitation at 559 nm, sequentially. Digital images were captured in a Confocal Laser Scanning Microscope (LSM-800, Zeiss). To quantify the number of BG4 foci, >50 cells were counted from at least three independent fields using ImageJ software.

18.0 Quantitative real-time PCR

Total RNA was extracted from the untreated and **TBP2** treated HeLa cells using TRIzol reagent (Invitrogen). The RNA extract was dissolved in DEPC treated water and precipitated with 75% ethanol to eliminate phenol contamination. RNA (1 μg/mL) was subjected to cDNA synthesis by the use of Verso cDNA synthesis kit (Thermo Scientific) as per the manufacturer's protocol. Real-time PCR was carried out in triplicate in which the following reaction mixtures were prepared to the indicated end concentration for each reaction: 4 μL of water, 3 μL of cDNA synthesis products, 0.5 μM of each primer for *MYC*, *BCL-2*, *c-KIT* or *18s* rRNA, and 7 μL of SYBR Green PCR Master Mix. Cycling conditions were 95 °C for 5 min, followed by 40 cycles of 95 °C for 15 s, 60 °C for 15 s, and 72 °C for 15 s. The calculation of relative gene expression was done using the $2^{-\Delta\Delta CT}$ method, in which the amount of *MYC* mRNA was normalized to an endogenous reference (18s rRNA).

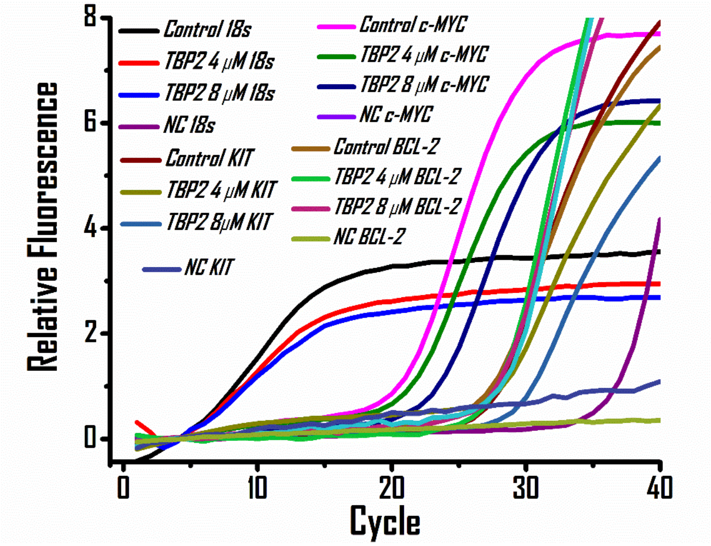

Fig. S12: Fluorescence curves for RNA quantification showing C_T values where 18S rRNA was used as the control gene to quantify the level of *c-MYC, BCL-2* and *c-KIT* gene expression. HeLa cells were treated at 4 μM and 8 μM of **TBP2**.

Table S1. Caculation of C_T values in TBP2 treated or untreated samples

**Sample (HeLa cells)**	**C_T value of 18s rRNA**	**C_T value of *c-MYC***	**ΔC_T value of *c-MYC***	**$\Delta\Delta C_T$ value of *c-MYC***	**$2^{-(\Delta\Delta C_T)}$ value of *c-MYC***
Untreated	7.1	18.72	11.62	0	1
**TBP2** (4 μM)	7.21	19.65	12.44	-0.82	0.56
**TBP2** (8 μM)	7.57	22.08	14.51	-2.89	0.14
**Sample**	**C_T value of 18s rRNA**	**C_T value of *BCL-2***	**ΔC_T value of *BCL-2***	**$\Delta\Delta C_T$ value of *BCL-2***	**$2^{-(\Delta\Delta C_T)}$ value of *BCL-2***
Untreated	7.1	27.15	20.05	0	1
**TBP2** (4 μM)	7.21	27.56	20.35	-0.30	0.81
**TBP2** (8 μM)	7.57	27.52	20	-0.05	0.97
**Sample**	**C_T value of 18s rRNA**	**C_T value of *c-KIT***	**ΔC_T value of *c-KIT***	**$\Delta\Delta C_T$ value of *c-KIT***	**$2^{-(\Delta\Delta C_T)}$ value of *c-KIT***
Untreated	7.1	26.99	19.89	0	1
**TBP2** (4 μM)	7.21	27.85	20.64	-0.75	0.94
**TBP2** (8 μM)	7.57	28.77	21.2	-1.3	0.41

Then, following equation was used to calculate the ΔC_T values:

$$\Delta C_T = C_{T\text{ target}} - C_{T\text{ referene}}$$

In our experiment, *c-MYC* and the 18s rRNA were the target and the reference gene, respectively.

Then, the $\Delta\Delta C_T$ values were calculated by:

$$\Delta\Delta C_T = \Delta C_{T\text{test sample}} - \Delta C_{T\text{calibrator sample}}$$

In our experiment, the ΔC_T values of the untreated are the calibrator samples and the ΔC_T values of the treated are test the sample.

Finally, the arithmetic calibrator was used to calculate the relative level of mRNA expression of the target genes:

$$2^{-(\Delta\Delta C_T)}$$

The qRT PCR was done using following primer sequences:

18S rRNA(forward): 5'-GATC$_2$GTG$_3$TG$_2$TG$_2$TGC-3'

18S rRNA (reverse): 5'-A$_2$GA$_2$GT$_2$G$_5$ACGC$_2$GA-3'

GAPDH (forward): 5'-GACG$_2$C$_2$GCATCT$_2$CT$_2$GT-3'

GAPDH (reverse): 5'-CACAC$_2$GAC$_2$T$_2$CAC$_2$AT$_4$-3'

c-KIT (forward): 5'-CGTG$_2$A$_4$GAGA$_4$CAGTCA-3'

c-KIT (reverse): 5'-CAC$_2$GTGATGC$_2$AGCTAT$_2$A-3'

c-MYC (forward):5'-CTGCGACGAG$_2$AG$_2$AG$_2$ACT-3'

c-MYC (reverse): 5'-G$_2$CAGCAGCTCGA$_2$T$_3$CT$_2$-3'

BCL-2 (forward): 5'-ACA$_2$CATG$_2$C$_3$TGTG$_2$ATGAC-3'

BCL-2 (reverse): 5'-T$_2$GT$_3$G$_4$CAG$_2$CATGT$_2$-3'

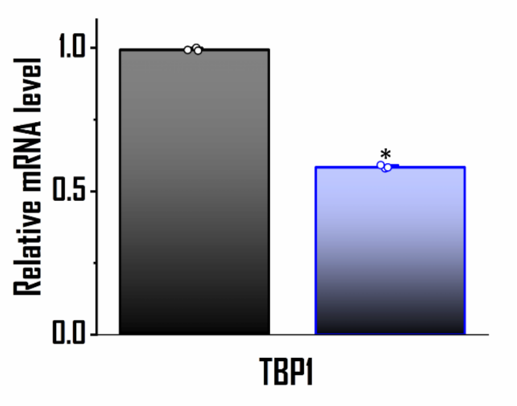

Fig. S13: qRT-PCR analysis for transcriptional regulation of *c-MYC* after treatment with **TBP1** in HeLa cells for 24 h. Quantification was done in terms of fold change by double delta C_T method using 18s rRNA as a housekeeping gene. Fold change of ligand treated relative gene expression is normalized with control. Three biological replicates were employed for the quantifications. Error bars represent mean ±SD.*P<0.05 (Student's t test), versus control HeLa cells.

19.0 Dual luciferase reporter assay

The luciferase reporter assay[25] was carried out by using Del4 *c-MYC* luciferase reporter plasmid procured from Addgene, USA and mutagenesis was accomplished by the Quick Change Site directed Mutagenesis kit (Agilent Technologies, CA, USA) with the following primers:

• Forward: 5'-*TGAG4CG2AGCTG2C2GCACG3AGA*-3'
• Reverse: 5'-*TCTC3GTGCG2C2AGCTC2GC4TCA*-3'

HeLa cells were cultured in DMEM media supplemented with 10% FBS and harvested in a six-well plate. The Del4 possesses 22-mer *c-MYC* G4 forming sequence in the P1 promoter upstream of the luciferase reporter (Addgene, USA). The G4 mutagenized Del4 plasmid was employed as a negative control. The G4 forming sequences upstream of luciferase reporter gene in the plasmids used in the current study are as following:

• Del4: 5'-*G4AG3TG4AG3TG4*-3'
• Mut-Del4: 5'-*G4AG3TG**AG2**AG3TG4*-3' (base substitution is in bold)

HeLa cells (~ 10^6 cells per well) were transfected with 1 μg plasmid using Lipofectamine 2000 as per manufacturer's instructions and co-transfected with renilla expressing luciferase plasmid pRL-TK. G4 forming sequences are not present in pRL-TK and could be used as positive control for transfection. After 6 h of incubation, cells were washed with PBS and fresh media was put in followed by treatment with **TBP2** (4 and 8 μM). Following 48 h of incubation, cells were lysed with 1X Cell Lysis buffer (Promega) with continuous pipetting and three cycles of vortexing for 30 seconds and stored at room temperature for 10 min. Luciferase assay was carried out in three biological replicates and luciferase expression was normalized by firefly to renila ratio and total protein concentration quantified by Lowry method.[26]

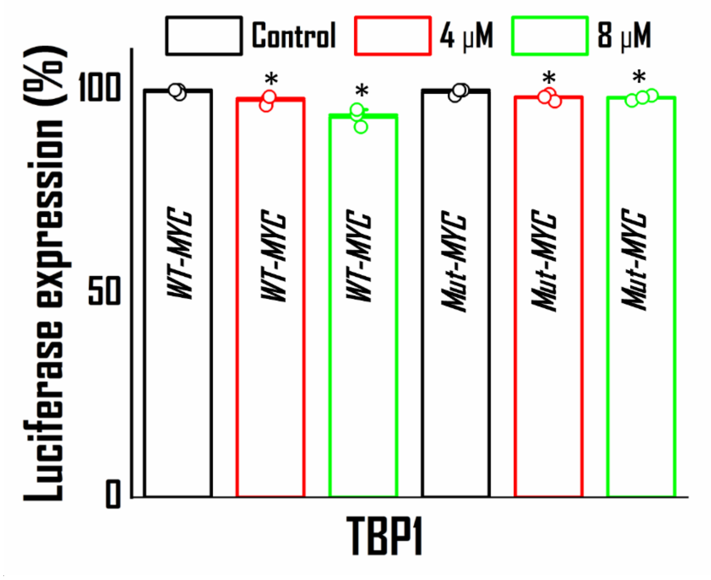

Fig. S14: Relative luciferase expression of *c-MYC* promoter normalized with the Renilla plasmid pRL-TK after treatment with **TBP1** at two different doses for 48 h. Percentage change of ligand treated relative luciferase expression is normalized with control or untreated HeLa cells. Error bars stand for mean ±SD. *P<0.05 (Student's t test), versus untreated HeLa cells.

20.0 UV absorption spectroscopy

UV measurements were carried out on a JASCO UV/Vis/NIR spectrophotometer at 25 °C in a thermo stated cell holder using a quartz cuvette with a 1 cm path-length. The absorption spectrum of **TBP1** and **TBP2** (10 μM) was recorded in 10 mM HEPES, 100 mM KCl or NaCl, pH 7.4.

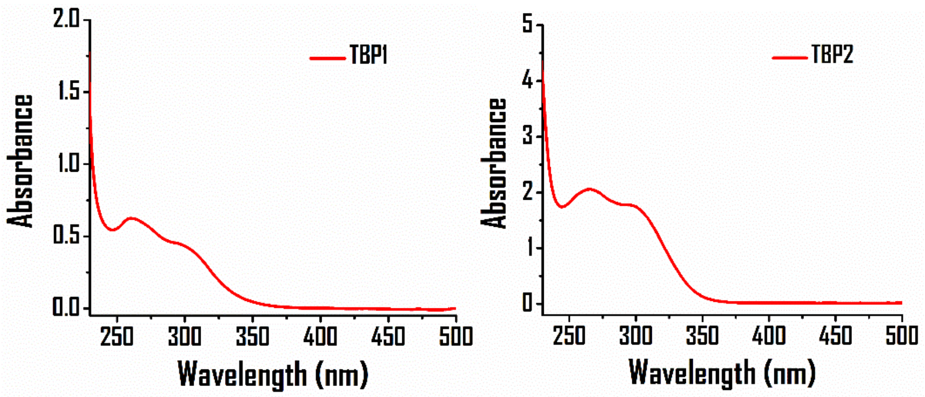

Fig. S15: UV-visible spectra of **TBP1** & **TBP2**.

21.0 Melting experiments by FRET

The stock solution of 100 μM 5′-FAM (Ex. 490 nm/Em. 520 nm) and 3′-TAMRA (Ex. 555 nm/Em. 580 nm) tagged DNA sequences were first diluted to 0.4 μM using a 60 mM potassium cacodylate (KCaco, pH 7.4) buffer. The diluted dual labeled oligonucleotides were annealed by heating to 95 °C for 1 min, then cooled slowly to room temperature and kept overnight at 4 °C. Subsequently, the DNAs (0.2 μM) were incubated with **TBP1** or **TBP2** (0 - 10 equivalent) in the same buffer at pH 7.4 for 1 h, using a blank 96-well plate (ThermoFisher Scientific) with a total volume of 100 μl for each well. In the presence of 60 mM K^+, the G4 DNA sequences mainly adopt a quadruplex structure in which the donor fluorophore FAM is in close proximity to the acceptor probe TAMRA resulting in a low FAM fluorescence due to the FRET effect. With increasing temperature, the conformation of G4 changes to a single-stranded form where the FAM is far from the TAMRA, which results in a high FAM fluorescence. Melting curves were then obtained by recording FAM fluorescence with increasing temperatures from 25 to 95 °C at a rate of 0.9 °C/min using Roche Light Cycler System 480II. The melting temperature (T_M)) values were determined by the maximum of the first derivative plot of the melting curves. The ΔT_M values were then plotted against the concentration of ligands to determine the stabilization potential for G4.

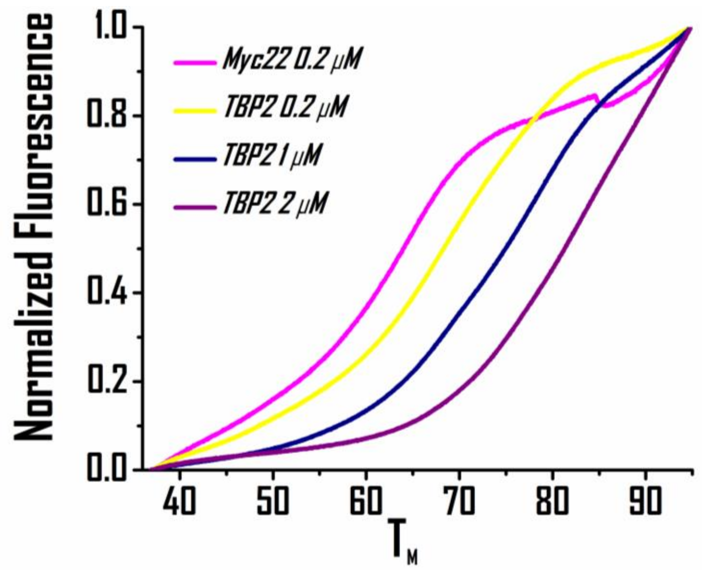

Fig. S16: FRET based experiments to measure *c-MYC22* G4 stabilization potential of **TBP2** at different concentrations.

22.0 Fluorescence spectroscopy

Fluorescence excitation and emission spectra were acquired with a HORIBA Jobin–Yvon FluoroMax-3 spectrofluorometer, in a 1 mm path length quartz cuvette at room temperature. Fluorescence of **TBP1** and **TBP2** was measured between 340 nm and 550 nm using the same excitation wavelength of 330 nm in degassed 60 mM KCaco buffer (pH 7.4). The titration experiments were carried out with the ligand **TBP1** and **TBP2** at 2.5 μM concentration in the presence of pre-annealed quadruplexes and *ds26* DNA. The specific concentrations of pre-annealed DNAs were added into the **TBP1** or **TBP2** solutions and incubated for 2 min before fluorescence measurement. The binding affinity or the apparent K_d was calculated by the Hill-1 equation using OriginPro 2016 software. The equation is as follows:

$$F = F_0 + \frac{(F_{max} - F_0)\,[DNA]}{K_d + [DNA]}$$

Where F signifies for fluorescence intensity, F_{max} for maximum fluorescence intensity, F_0 for fluorescence intensity in the absence of DNA and K_d for dissociation constant. Following DNA sequences were used for fluorescence titration:

c-MYC22: 5'-d(G3GAG3TG4AG3TG4)-3'

BCL-2: 5'-d(G3CGCG3AG2A2TTG3CG3)-3'

c-KIT1: 5'-d(G3AG3CGCTG3AG2AG3)-3'

c-KIT2: 5'-d(G3CG3CGCTAG3AG4)-3'

h-TELO: 5’-d(T2G3T2AG3T2AG3AT2AG3A)-3'

ds26 DNA: 5'-d(TATAGCTATA8TATAGCTATA)-3'

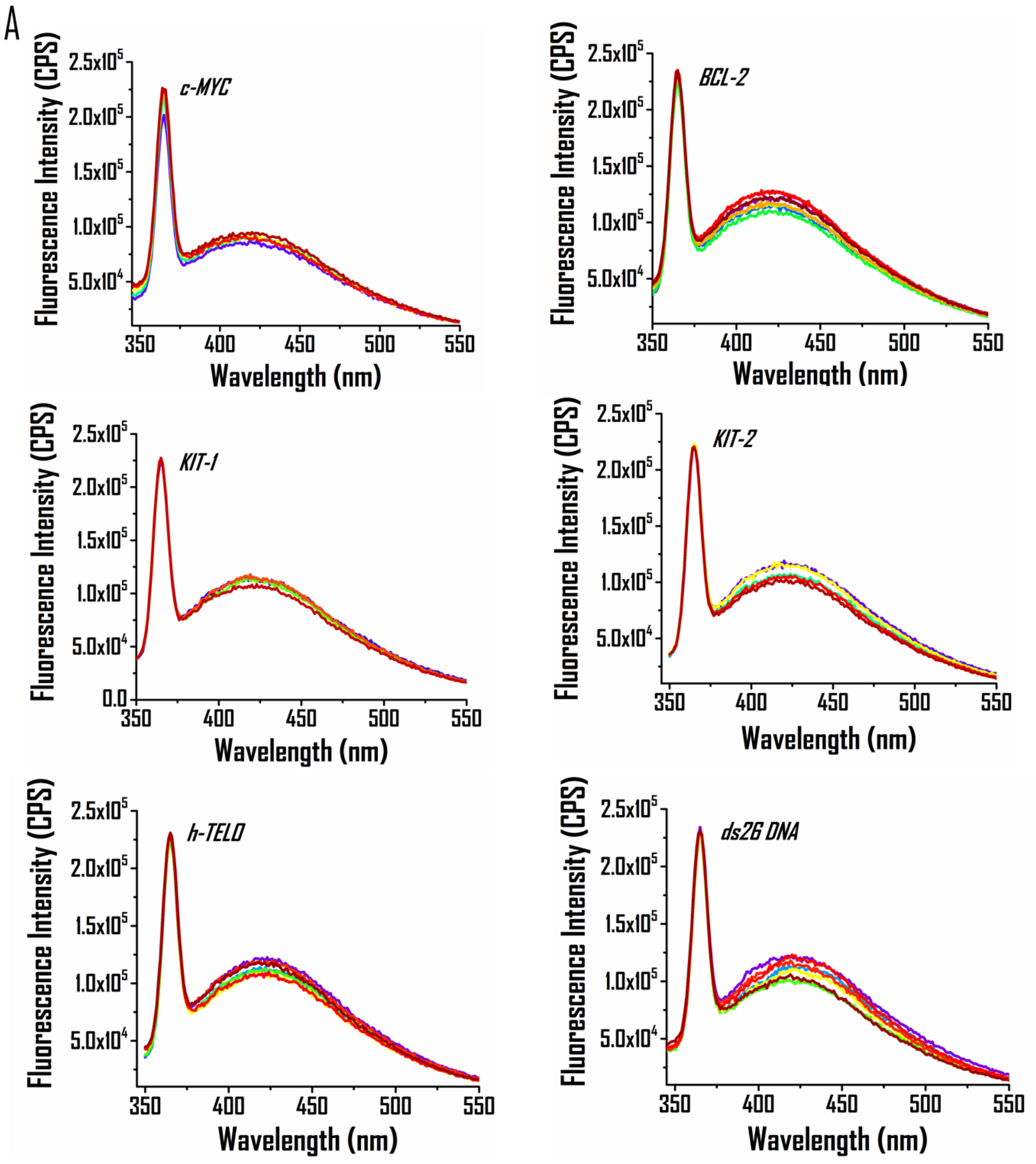
A
c-MYC
BCL-2
KIT-1
KIT-2
h-TELO
ds26 DNA
Fluorescence Intensity (CPS)
2.5x10^5
2.0x10^5
1.5x10^5
1.0x10^5
5.0x10^4
0.0
Wavelength (nm)
350
400
450
500
550

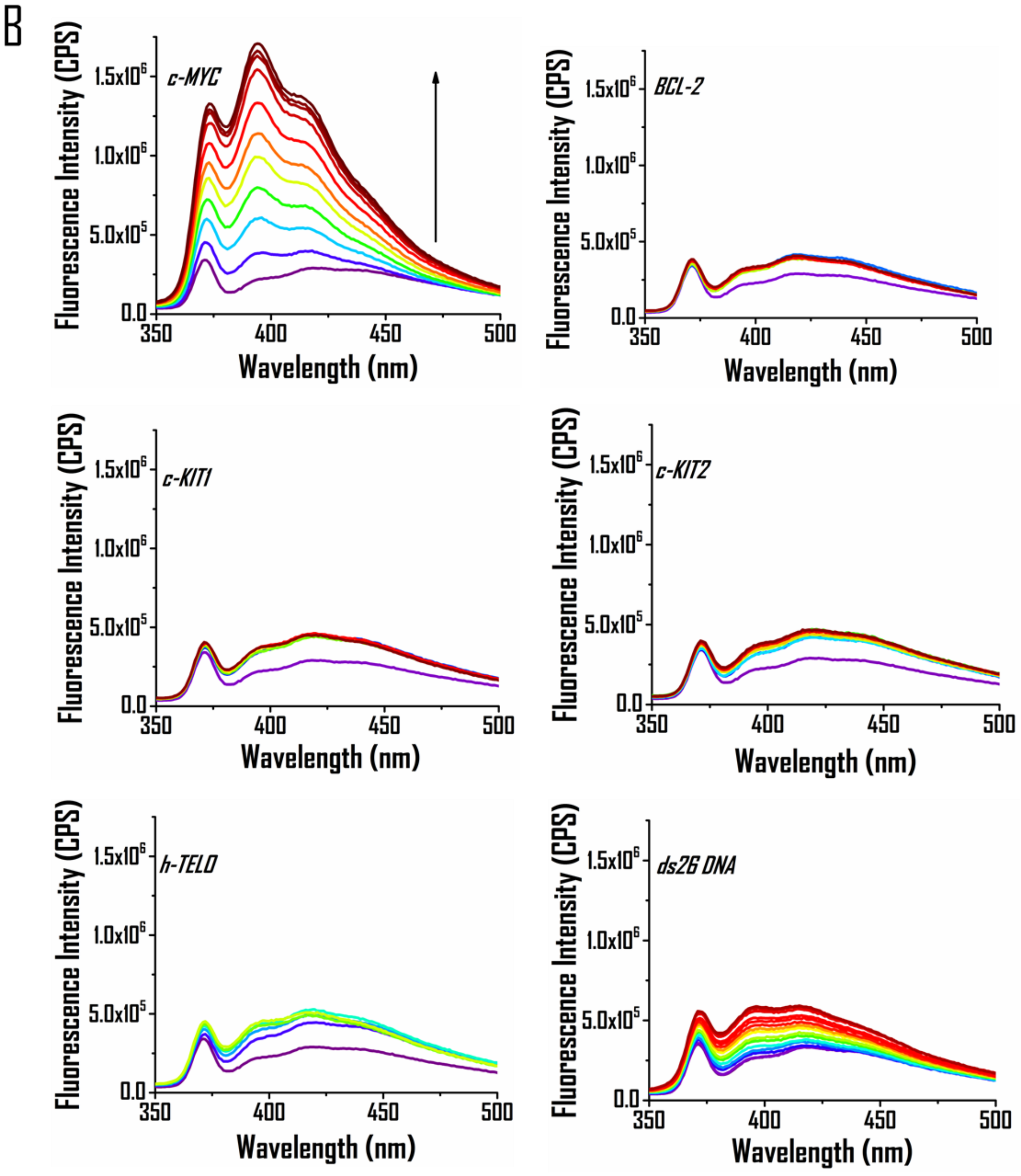

Fig. S17: Fluorometric titration of **(A) TBP1** and **(B) TBP2** with G4s (*c-MYC22*, *BCL-2*, *c-KIT1*, *c-KIT2*, *h-TELO*) and *ds26* DNA in 60 mM potassium cacodylate buffer (pH 7.4).

23.0 NMR titration

The *c-MYC22* DNA was purchased from Eurofins MWG Operon in HPSF grade followed by purification with HPLC. The titration was carried out with 100 μM DNA in 25 mM Tris•HCl buffer (pH 7.4), which contains 100 mM KCl in 5% d6-DMSO/95% H_2O. Low volume of **TBP2** stock solution in 100% d6-DMSO was added directly into the NMR tube (7.5% d6-DMSO at the end of the titration). 2,2-dimethyl-2-silapentane-5-sulphonate (DSS) was kept as reference. For water suppression, gradient-assisted excitation sculpting or jump-return-Echo was used.[22-24]

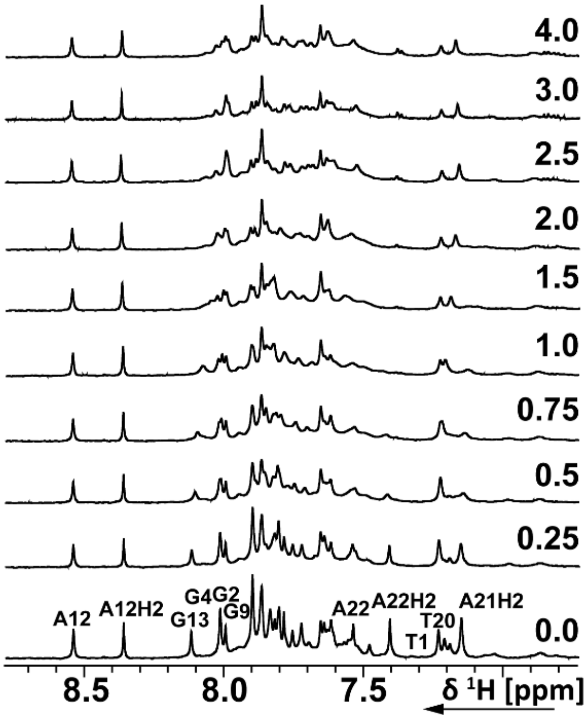

Fig. S18: 1D ^{1}H NMR spectra of the aromatic region of *c-MYC22* in 25 mM Tris·HCl buffer containing 100 mM KCl at different [**TBP2**:DNA] molar ratios.

References

1. A. Gilles, M. Barboiu, Highly selective artificial K^+ channels: an example of selectivity-induced transmembrane potential. *J. Am. Chem. Soc.* **138**, 426-432 (2016).
2. Y. P. Kumar, R. N. Das, S. Kumar, O. M. Schütte, C. Steinem, J. Dash, Triazole-Tailored Guanosine Dinucleosides as Biomimetic Ion Channels to Modulate Transmembrane Potential. *Chem. Eur. J.* **20**, 3023-3028 (2014).
3. Malla, J. A.; Umesh, R. M.; Vijay, A.; Mukherjee, A.; Lahiri, M.; Talukdar, P., Apoptosis-inducing activity of a fluorescent barrel-rosette M+/Cl− channel. *Chem. Sci.* **11**, 2420-2428 (2020).

4. C. Lee, W. Yang, R. G. Parr, Development of the Colle-Salvetti correlation-energy formula into a functional of the electron density. *Phys. Rev. B* **37**, 785-789 (1988).
5. Y. Zhao, D. G. Truhlar, The M06 suite of density functionals for main group thermochemistry, thermochemical kinetics, noncovalent interactions, excited states, and transition elements: two new functionals and systematic testing of four M06-class functionals and 12 other functionals. *Theor. Chem. Acc.* **120**, 215-241 (2008).
6. P. C. Hariharan, J. A. Pople, The influence of polarization functions on molecular orbital hydrogenation energies. *Theor. Chim. Acta* **28**, 213-222 (1973).
7. V. A. Rassolov, J. A. Pople, M. A. Ratner, T. L. Windus, 6-31G* basis set for atoms K through Zn. *J. Chem. Phys.* **109**, 1223-1229 (1998).
8. T. K. Mukhopadhyay, K. Bhattacharyya, A. Datta, Gauging the Nanotoxicity of h2D-C2N toward Single-Stranded DNA: An in Silico Molecular Simulation Approach. *ACS Appl. Mater. Interfaces* **10**, 13805-13818 (2018).
9. T. K. Mukhopadhyay, A. Datta, Screening two dimensional materials for the transportation and delivery of diverse genetic materials. *Nanoscale* **12**, 703-719 (2020).
10. T. K. Mukhopadhyay, A. Datta, Delicate Balance of Non-Covalent Forces Govern the Biocompatibility of Graphitic Carbon Nitride towards Genetic Materials. *ChemPhysChem* **21**, 1836-1846 (2020).
11. E. Darve, D. Rodríguez-Gómez, A. Pohorille, Adaptive biasing force method for scalar and vector free energy calculations. *J. Chem. Phys.* **128**, 144120 (2008).
12. S. E. Feller, Y. Zhang, R. W. Pastor, B. R. Brooks, Constant pressure molecular dynamics simulation: The Langevin piston method. *J. Chem. Phys. 103*, 4613-4621 (1995).
13. R. L. Davidchack, R. Handel, M. V. Tretyakov, Langevin thermostat for rigid body dynamics. *J. Chem. Phys*. **130**, 234101 (2009).
14. O. Farago, Langevin thermostat for robust configurational and kinetic sampling. *Phys. A: Stat. Mech. Appl.* **534**, 122210 (2019).
15. H. C. Andersen, Rattle: A "velocity" version of the shake algorithm for molecular dynamics calculations. *J. Comput. Phys.* **52**, 24-34 (1983).
16. L. Kalé, R. Skeel, M. Bhandarkar, R. Brunner, A. Gursoy, N. Krawetz, J. Phillips, A. Shinozaki, K. Varadarajan, K. Schulten, NAMD2: Greater Scalability for Parallel Molecular Dynamics. *J. Comput. Phys.* **151**, 283-312 (1999).
17. W. Humphrey, A. Dalke, K. Schulten, VMD: Visual molecular dynamics. *J. Mol. Graph.* **14**, 33-38 (1996).
18. K. Vanommeslaeghe, E. Hatcher, C. Acharya, S. Kundu, S. Zhong, J. Shim, E. Darian, O. Guvench, P. Lopes, I. Vorobyov, A. D. Mackerell Jr, CHARMM general force field: A force field for drug-like molecules compatible with the CHARMM all-atom additive biological force fields. *J. Comput. Chem.* **31**, 671-690 (2010).
19. W. Yu, X. He, K. Vanommeslaeghe, A. D. MacKerell Jr, Extension of the CHARMM general force field to sulfonyl-containing compounds and its utility in biomolecular simulations. *J. Comput. Chem.* **33**, 2451-2468 (2012).
20. W. L. Jorgensen, J. Chandrasekhar, J. D. Madura, R. W. Impey, M. L. Klein, Comparison of simple potential functions for simulating liquid water. *J. Chem. Phys.* **79**, 926-935 (1983).
21. C. Møller, M. S. Plesset, Note on an Approximation Treatment for Many-Electron Systems. *Phys. Rev.* **46**, 618-622 (1934).
22. Complete reference for Gaussian09

    Gaussian 09; M. J. Frisch, G. W. Trucks, H. B. Schlegel, G. E. Scuseria, M. A. Robb, J. R. Cheeseman, G. Scalmani, V. Barone, B. Mennucci, G. A. Petersson, H. Nakatsuji, M. Caricato, X. Li, H. P. Hratchian, A. F. Izmaylov, J. Bloino, G. Zheng, J. L. Sonnenberg, M. Hada, M. Ehara, K. Toyota, R. Fukuda, J. Hasegawa, M. Ishida, T. Nakajima, Y. Honda, O. Kitao, H. Nakai, T. Vreven, J. A. Montgomery, Jr., J. E. Peralta, F. Ogliaro, M. Bearpark, J. J. Heyd, E. Brothers, K. N. Kudin, V. N. Staroverov, T. Keith, R. Kobayashi, J. Normand, K. Raghavachari, A. Rendell, J. C. Burant, S. S. Iyengar, J. Tomasi, M. Cossi, N. Rega, J. M. Millam, M. Klene,

J. E. Knox, J. B. Cross, V. Bakken, C. Adamo, J. Jaramillo, R. Gomperts, R. E. Stratmann, O. Yazyev, A. J. Austin, R. Cammi, C. Pomelli, J. W. Ochterski, R. L. Martin, K. Morokuma, V. G. Zakrzewski, G. A. Voth, P. Salvador, J. J. Dannenberg, S. Dapprich, A. D. Daniels, O. Farkas, J. B. Foresman, J. V. Ortiz, J. Cioslowski, and D. J. Fox, *Gaussian, Inc.*, Wallingford CT (2010).

23. A. Ambrus, D. Chen, J. Dai, R. A. Jones, D. Yang, Solution structure of the biologically relevant G-quadruplex element in the human c-MYC promoter. Implications for G-quadruplex stabilization. *Biochemistry* **44**, 2048-2058 (2005).
24. V. Sklenář, A. Bax, Spin-echo water suppression for the generation of pure-phase two-dimensional NMR spectra. *J. Magn. Reson.* **74**, 469-479 (1987).
25. T.-L. Hwang, A. Shaka, Water suppression that works. Excitation sculpting using arbitrary wave-forms and pulsed-field gradients. *J. Magn. Reson.* **112**, 275-279 (1995).
26. D. Dutta, M. Debnath, D. Müller, R. Paul, T. Das, I. Bessi, H. Schwalbe, J. Dash, Cell penetrating thiazole peptides inhibit c-MYC expression via site-specific targeting of c-MYC G-quadruplex. *Nucleic Acids Res.* **46**, 5355-5365 (2018).
27. L. OH, Lowry method, a modification of the Folin method based on the presence of tyrosine and tryptophan in proteins. *J Biol Chem* **193**, 265-266 (1951).
