# Supplementary material for: A non-B DNA binding peptidomimetic channel alters cellular functions
Source: Nat Commun. 2024 Jun 20;15:5275. doi: 10.1038/s41467-024-49534-0 (PMC11190219; doi:10.1038/s41467-024-49534-0)
Supplement: Supplementary file 3 — Description of Additional Supplementary Files [file 41467_2024_49534_MOESM3_ESM.pdf]

Description of Additional Supplementary Files

File Name: Supplementary Movie 1

Description: *MD simulation movie of Ion Transport via **TBP2** Gated Channel*

The movie provided along with the supporting information depicts the MD simulation representing passage of a sodium ion through the supramolecular assembly of **TBP2** molecules, embedded inside a POPC lipid membrane. The lipid molecules are not shown for clarity of representation. The sodium ion enters the ion channel; moves between successive **TBP2** molecules stacked upon each other and eventually get released.
